# Molecular mechanisms of heterosis under drought stress in maize hybrids Zhengdan7137 and Zhengdan7153

**DOI:** 10.3389/fpls.2024.1487639

**Published:** 2024-10-08

**Authors:** Kai Dai, Zhanyi Zhang, Sen Wang, Jiwei Yang, Lifeng Wang, Tengjiao Jia, Jingjing Li, Hao Wang, Song Song, Yuncai Lu, Huiyong Li

**Affiliations:** ^1^ Institute of Crop Germplasm Resources, Henan Academy of Agricultural Sciences, Zhengzhou, China; ^2^ College of Advanced Agriculture and Ecological Environment, Heilongjiang University, Haerbin, China; ^3^ College of Life Sciences, Henan Agricultural University, Zhengzhou, China

**Keywords:** drought, re-watering, heterosis, maize, RNA-Seq, overdominant genes, underdominant genes

## Abstract

Maize is one of the most successful crops in utilizing heterosis which significantly improves maize stresses resistance and yield. Drought is a destructive abiotic stress that significantly reduces crop yield, particularly in maize. Drought stress and re-watering frequently occur during the growth and development of maize; however, the molecular mechanisms of heterosis under drought stress and re-watering have rarely been systematically investigated. Zhengdan7137 and Zhengdan7153 are two maize hybrid varieties with robust heterosis, and separately belongs to the SS×NSS and Reid×Tangsipingtou heterotic groups. 54 transcriptomes of these two hybrids and their parental inbred lines were analyzed under well-watering (WW), water-deficit (WD), and re-watering (RW) conditions using RNA-Seq. In this study, we identified 3,411 conserved drought response genes (CDRGs) and 3,133 conserved re-watering response genes (CRRGs) between Zhengdan7137 and Zhengdan7153. When comparing CDRGs and CRRGs to overdominance and underdominance genes, we identified 303 and 252 conservative drought response overdominance genes (DODGs) and underdominance genes (DUDGs), respectively, and 165 and 267 conservative re-watering response overdominance genes (RODGs) and underdominance genes (RUDGs), respectively. DODGs are involved in stress response-related processes, such as L-phenylalanine metabolism, carbohydrate metabolism, and heat response, whereas DUDGs are associated with glucose metabolism, pentose-phosphate shunt, and starch metabolism. RODGs and RUDGs contribute to the recovery of hybrids from drought stress by upregulating cell propagation and photosynthesis processes, and repressing stress response processes, respectively. It indicated overdominant and underdominant genes conservatively contributed to hybrid heterosis under drought stress. These results deepen our understanding of the molecular mechanisms of drought resistance, uncover conservative molecular mechanisms of heterosis under drought stress and re-watering, and provide potential targets for improving drought resistance in maize.

## Introduction

Maize (*Zea mays L.*) is the main cereal crop planted worldwide and used as food, livestock forage, and industrial material. Drought is a typical destructive abiotic stress that systematically damages maize organs and physiological functions, leading to significant yield losses (Tari and Fathi, 2016). Moreover, the risk of drought has increased significantly with global warming. In recent years, extreme long-term droughts accompanied by high temperatures have frequently occurred worldwide, threatening maize agricultural production (Sheoran et al., 2022). Therefore, improving the drought resistance of maize is an urgent task to ensure global food safety and satisfy industrial demands.

Drought, a water-deficit condition, primarily causes plant cellular dehydration and an increase in osmotic pressure, resulting in the induction of a series of physiological and biochemical reactions such as plasma membrane disruption, photosynthesis reaction inhibition, and excessive reactive oxygen species (ROS) accumulation which promote lipid peroxidation and macromolecule (e.g., protein, lipid, and nucleic acid) degradation (Singh et al., 2023; Liu et al., 2023). Ultimately, drought can lead to the impairment of plant cell vitality and even cell death. And plants exhibit various drought stress phenotypes depending on the developmental stage, such as stunted seedling growth, rolling leaves, pollen abortion, enlarged anthesis-silking interval (ASI), seed-filling suppression, and grain yield loss (Liu et al., 2023; Hu et al., 2020; Zenda et al., 2018; Bheemanahalli et al., 2022). The grain yield is the primary trait used to assess drought impairment in crops. Secondary traits, e.g., 100-kernel weight (KW), are also used as criteria for the evaluation of drought impairment (Monneveux et al., 2008). To cope with drought stress, plants activate a range of morphological, physiological, and biochemical mechanisms known as drought resistance, to preserve their viability and reproduction (Liu et al., 2023). Drought resistance is involved in maintaining cellular homeostasis to adapt to increased osmotic pressure through the synthesis of osmoprotectants, activating ROS scavenging, adjusting regulatory pathways of phytohormones [e.g., abscisic acid (ABA), auxin and ethylene], and expressing more molecular chaperones (Liu et al., 2023; Singh et al., 2023; Sheoran et al., 2022; Waititu et al., 2021). Damage caused by drought stress can be restored by re-watering (Wang et al., 2022b). Plants can then undergo extraordinarily rapid growth to compensate or overcompensate for the losses incurred during drought stress. Therefore, the capacity of plants to recover after re-watering is a criterion for evaluating drought resistance.

Heterosis is a phenomenon widely used in crop breeding, in which hybrids exhibit superior performance compared to their homozygous parents (e.g. higher biomass, developmental speed, yield, and resistance to environmental stresses such as drought) (Luo et al., 2021). Therefore, elucidating the mechanisms of heterosis is important (Lippman and Zamir, 2007). Multiple hypotheses have been formed to explain the genetic basis of heterosis (including dominance, overdominance, epistasis). Recently, homo-insufficiency under insufficient background (HoIIB) has also been proposed (Hochholdinger and Baldauf, 2018; Xie et al., 2022). However, these genetic hypotheses have not revealed the molecular mechanisms underlying heterosis. With advancements in high-throughput transcriptome profiling technology, differentially expressed genes (DEGs) between hybrids and their parental lines have been identified and found to be responsible for heterosis (Li et al., 2015). The DEGs can be classified into additive genes and nonadditive genes consisting of dominant, overdominant, and underdominant genes. These different expression patterns genes contribute differently to heterosis depending on the species, genetic background, and environment. For examples, additive genes have been suggested to be fundamental for maize development heterosis (Zhao et al., 2019). Nonadditive genes are associated with heterosis, particularly under environmental stresses such as heat, drought, and salt (Zhao et al., 2019; Marcon et al., 2017; Zhang et al., 2022a; Liu et al., 2024). In rubber tree seedlings, over-dominant genes play an important role in growth heterosis (Yang et al., 2018).

Unveiling the molecular mechanisms that underlie heterosis in maize hybrids under drought and re-watering stresses will offer novel insights and genes for drought resistance, which can significantly contribute to drought resistance improvement. However, such investigations have been sparingly undertaken. Zhengdan7137 (Zheng1110×Zheng1117) and Zhengdan7153 (Zheng1121×Zheng641) are two hybrid varieties of maize with significant heterosis under normal and drought conditions, and separately belongs to the SS×NSS and Reid×Tangsipingtou heterotic groups. The SS×NSS and Reid×Tangsipingtou are two typical and widely used heterosis patterns in China. To explore the molecular mechanisms of heterosis under drought and re-watering stresses, we analyzed the transcriptomes of Zhengdan7137, Zhengdan7153, and their parental inbred lines to explore the molecular mechanisms of heterosis under well-watered (WW), water-deficit (WD), and re-watering (RW) conditions. For convenience, Zhengdan7137 and its parental inbred lines Zheng1110 and Zheng1117 were designated as AB, AA, and BB, respectively, whereas Zhengdan7153 and its parental inbred lines Zheng1121 and Zheng641 were designated as CD, CC, and DD, respectively. Our results revealed the conserved molecular mechanisms of drought resistance and heterosis under drought stress.

## Results

### Heterosis of hybrids Zhengdan7137 and Zhengdan7153 under drought stress

**Figure 1:**
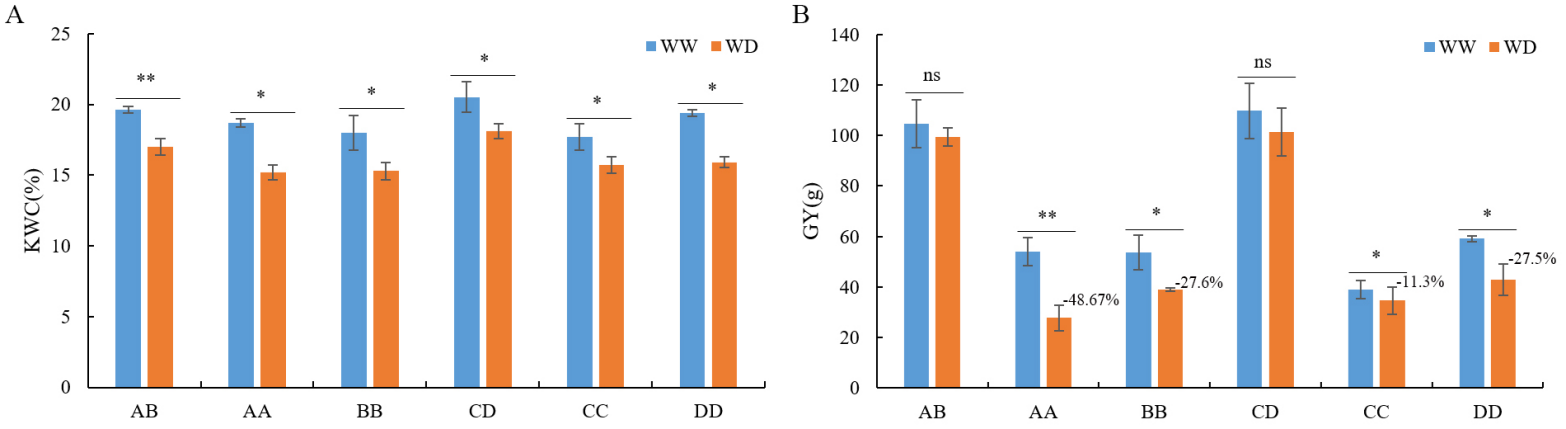
Kernels water content **(A)** and grain yield per ear **(B)** of AB, CD and their parental inbred lines under well-watering (WW) and water-deficit (WD) conditions. KWC, kernels water content; GY, grain yield per ear; “*”, “**” and ns, p-value<0.05, <0.01 and >0.05 in student’s t test, respectively. AB, hybrid Zhengdan7137; AA, inbred line Zheng1110; BB, inbred line Zheng1117. AA and BB are parental inbred lines of AB. CD, hybrid Zhengdan7153; CC, inbred line Zheng1121; DD, inbred line Zheng641. CC and DD are parental inbred lines of CD.

To ascertain the impact of drought on heterosis, we cultivated the hybrids AB, CD, and their parental inbred lines under WW and WD conditions and investigated traits including length, thickness, and grain yield of each ear (EL, ET, and GY, respectively), 100-kernel weight (KW), and kernel water content (KWC) to evaluate heterosis. Compared with WW conditions, the KWC of AB, CD, and their parental inbred lines significantly decreased under WD conditions, which corresponded to drought stress (**Figure 1A**). Under WW and WD conditions, AB and CD consistently displayed significantly superior performance than their parents in EL, ET, KW, GY, and KWC with varied mid-parent heterosis (MPH, 6.9–198.1%) and high-parent heterosis (HPH, 10.0–155.5%), except for KWC under WW conditions of AB and CD, KW of AB under WW conditions, and KW of CD under WD conditions (**Supplementary Table S1**). In addition, the GY of AB and CD displayed the highest MPH (94.1–198.1%) and HPH (85.5–155.5%) (**Supplementary Table S1**). Hence, hybrids AB and CD exhibited reliable heterosis under both normal and drought conditions.

Compared to WW conditions, the EL, ET, and GY of hybrids AB and CD were not significantly altered under WD conditions, whereas the GY of the parental inbred lines, EL, ET, and KW of AA, ET of BB, EL of CC, and ET of DD were significantly decreased (**Figure 1B**; **Supplementary Table S1**). In contrast, the KW of AB, CD, and CC increased under drought conditions owing to the lower seed-setting rate (**Supplementary Table S1**). Moreover, under WD condition, the GY in AA, BB, CC, and DD decreased by 48.6%, 27.6%, 11.3%, and 27.5%, respectively, which were significantly higher than hybrids AB and CD (**Figure 1B**). The GY is the primary criterion used for evaluating drought impairment (Monneveux et al., 2008). Therefore, these results indicated that hybrids AB and CD possessed higher drought resistance than their parent inbred lines; in other words, AB and CD exhibited drought-resistant heterosis.

### RNA-Seq of hybrids Zhengdan7137, Zhengdan7153 and their parental inbred lines

**Figure 2:**
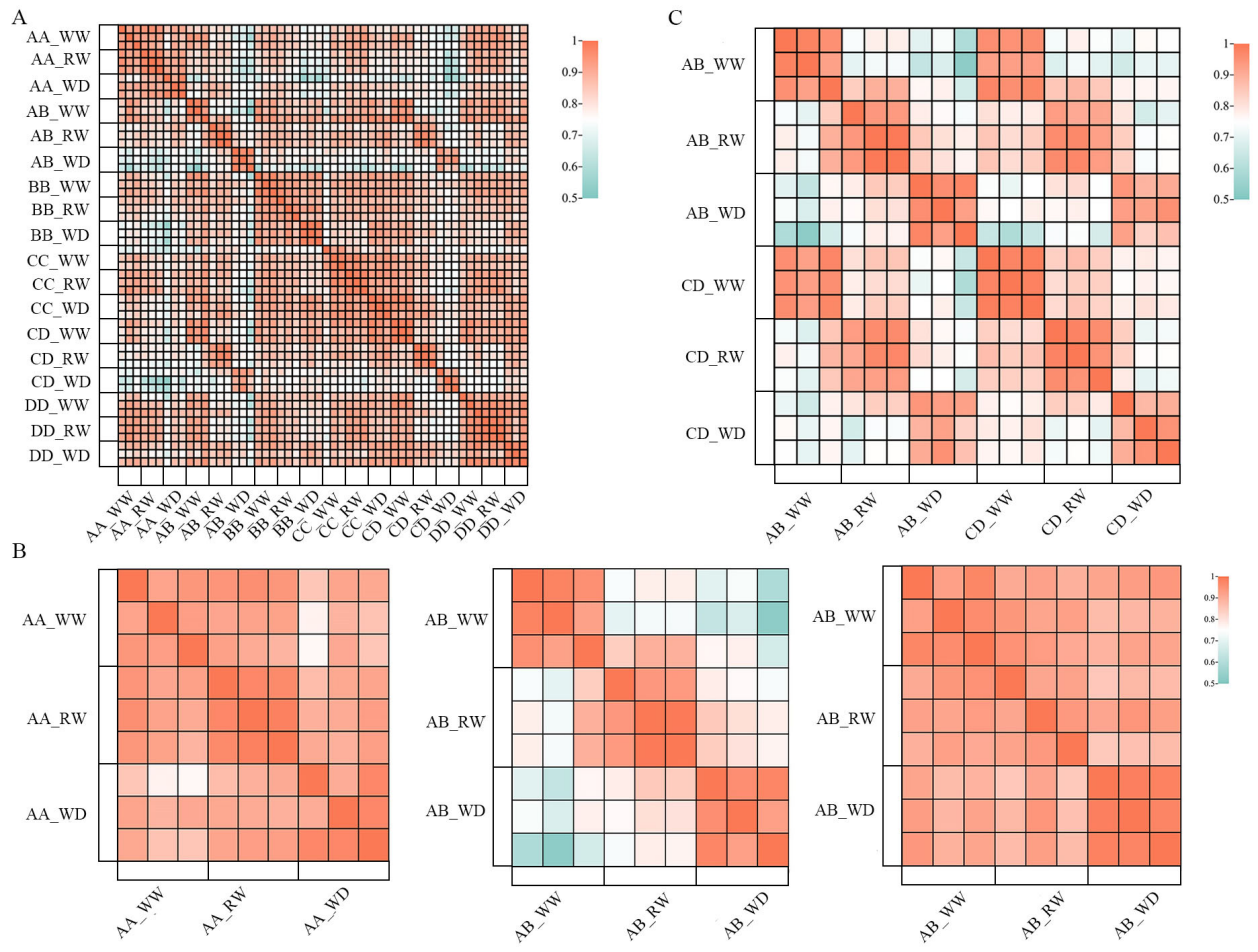
The Pearson correlation rate analysis of RNA-Seq libraries of AB, CD and their parental inbred lines under WW, WD and RW conditions. **(A)** the biological repeates of RNA-Seq libraries were high related in the Pearson correlation rate ananlysis; **(B)** Pearson correlation rates between WW, WD and RW conditions of hybrid AB (middle) were significant lower than its parental inbred lines AA (left) and BB (right); **(C)** RNA-Seq librarys of hybrids AB and CD were highly correlated under WW, WD and RW conditions. AB, hybrid Zhengdan7137; AA, inbred line Zheng1110; BB, inbred line Zheng1117. AA and BB are parental inbred lines of AB. CD, hybrid Zhengdan7153; CC, inbred line Zheng1121; DD, inbred line Zheng641. CC and DD are parental inbred lines of CD.

To investigate the molecular mechanism of heterosis under drought and re-watering stress, we analyzed the transcriptomes of the leaves of hybrids AB, CD, and their parental inbred lines with three biological repeats under WW, WD, and RW conditions. Fifty-four RNA-seq libraries were constructed and a total of 349.32 G clean data were obtained after quality control, in which each clean library was 5.84–7.46G. The Q30, Validbases, and GC contents of clean reads from each library were 91.8–93.5%, 93.9–99.1%, and 51.9–55.8%, respectively. On average, 85.6% of the clean reads were uniquely mapped to the reference genome B73 (**Supplementary Table S2**). In addition, the biological replicates were highly reproducible (Pearson’s correlation rate > 0.88) (**Figure 2A**). These results indicate the high quality of our transcriptome data for AB, CD, and their parental inbred lines. The Pearson correlation rates between the WW, WD, and RW conditions of hybrids AB and CD were significantly lower than those of their parental inbred lines, suggesting that the transcriptomes of the hybrids might be more sensitive to drought and re-watering stresses than their parental inbred lines (**Figure 2B**). Hybrids AB and CD were highly correlated (R > 0.83) under WW, WD, and RW conditions (**Figure 2C**), suggesting that there might be a large similarity between hybrids AB and CD in dealing with drought and re-watering stresses. Overall, our transcriptome data were ideal for investigating the molecular mechanisms underlying drought resistance and heterosis.

### Drought response genes of hybrids Zhengdan7137, Zhengdan7153, and their parental inbred lines

**Figure 3:**
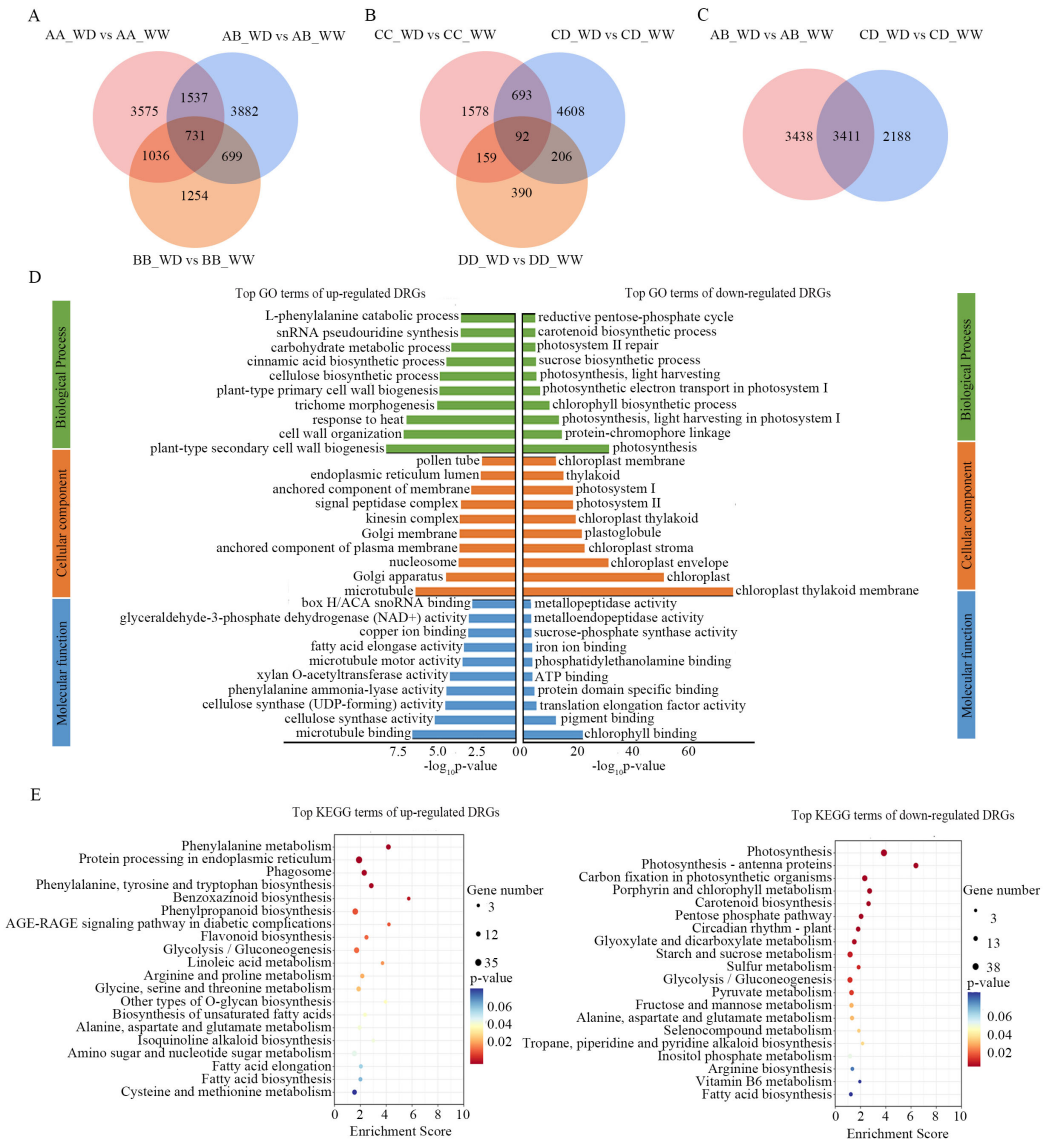
Drought response genes of hybrids AB, CD and their parental inbred lines. **(A, B)** venn diagrams of drought response genes in AB, AA and BB **(A)**, and CD, CC, and DD **(B)**; **(C)** venn diagrams of drought response genes of AB and CD; **(D)**, top 30 enrichment terms in GO analysis of up- and down- regulated CDRGs; **(E)**, top 20 enrichment terms in KEEG pathways analysis of up- (left) and down- (right) regulated CDRGs. AB, hybrid Zhengdan7137; AA, inbred line Zheng1110; BB, inbred line Zheng1117. AA and BB are parental inbred lines of AB. CD, hybrid Zhengdan7153; CC, inbred line Zheng1121; DD, inbred line Zheng641. CC and DD are parental inbred lines of CD.

We identified differentially expressed genes (|fold change| > 2 and FDR < 5%) in response to drought stress in the two hybrids AB, CD, and their parental inbred lines based on six pairwise comparisons between the WW and WD conditions (AA_WD vs. AA_WW, BB_WD vs. BB_WW, AB_WD vs. AB_WW, CC_WD vs. CC_WW, DD_WD vs. DD_WW, and CD_WD vs. CD_WW), which are referred to as drought response genes (DRGs). There were more DRGs in AB, AA, and BB (12,714) than in CD, CC, and DD (7,726). Hybrids AB (6,849) and CD (5,579) had more DRGs than their parental inbred lines BB (3,720), CC (2,522), and DD (847), but not AA (6,879) (**Figures 3A, B**). There were 731, 2,268, 1,430 and 1,767 common DRGs in AA/BB/AB, AA/AB, BB/AB, and AA/BB, respectively. Moreover, 3,882, 3,575, and 1,254 DRGs were unique in AB, AA, and BB, respectively. Common DRGs (92, 785, 298, and 251 in CD/CC/DD, CD/CC, CD/DD, and CC/DD, respectively) were less than those in AB. The number of unique DRGs in CD (4,608) was significantly higher than in CC (1,578) and DD (390) (**Figures 3A, B**). The unique DRGs in AB and CD are potential molecular bases for the drought-resistant heterosis. Notably, there were 3,411 common DRGs between AB and CD, accounting for 49.8% and 60.9% of the DRGs of AB and CD, respectively (**Figure 3C**), which were referred to as conservative DRGs (CDRGs), suggesting that there were similar molecular mechanisms for hybrids to deal with drought stress. Among the CDRGs, 26 genes were common to all parental inbred lines, including *Bx1* which is known to be dramatically upregulated under drought stress in maize (Waititu et al., 2021), and *Pip2f*, which encodes a water channel protein that might be involved in water transport (Lopez et al., 2003).

The CDRGs contained 1,751 upregulated genes and 1660 downregulated genes. To explore the roles of CDRGs, Gene Ontology (GO) functional classification and Kyoto Encyclopedia of Gene and Genomes (KEGG) pathway analyses were performed for the upregulated and downregulated CDRGs. We found that drought stress significantly inhibited photosynthesis, as evidenced by the downregulation of CDRGs significantly enriched in photosynthesis-related terms (e.g., photosynthesis, protein-chromophore linkage, light harvesting in photosystem I in GO; photosynthesis, and photosynthesis-antenna proteins in KEGG) (**Figures 3D, E**). The upregulated CDRGs were significantly enriched in stress response-related GO terms, such as plant-type secondary cell wall biogenesis, response to heat, cinnamic acid biosynthetic process, carbohydrate metabolic process, and L-phenylalanine catabolic process (**Figure 3D**). In addition, these upregulated CDRGs were enriched in KEGG pathways correlating with osmolality (e.g., arginine and proline metabolism, alanine, aspartate, and glutamate metabolism, glycine, serine, and threonine metabolism, and amino sugar and nucleotide sugar metabolism) and antioxidant capacity (e.g., flavonoid biosynthesis and phenylpropanoid biosynthesis) (**Figure 3E**). These results indicated that the hybrids adjusted multiple biological processes and metabolic pathways to maintain cell stability and cope with drought stress predominantly by upregulating stress response genes.

### Re-watering response genes of hybrids Zhengdan7137, Zhengdan7153, and their parental inbred lines

In parallel, we identified differentially expressed genes (|fold change| > 2 and FDR < 5%) in response to re-watering stress in AB, CD, and their parental inbred lines by comparing RW to WD conditions, referred to as re-watering response genes (RRGs). There were 6,096 and 5,388 RRGs in AB and CD, respectively, which were higher than those in BB (3,433), CC (1,933), and DD (3,052), but not in AA (7,000). In addition, 3,355 (55%) and 3,924 (72.8%) RRGs were unique in hybrids AB and CD, respectively (**Supplementary Figures S1A, B**). We identified 3,133 conserved RRGs (CRRGs) that were common between AB and CD (**Supplementary Figure S1C**). A total of 160 RRGs were common between the two hybrids and their inbred lines. In the CRRGs, 1,415 and 1,718 genes were upregulated and downregulated, respectively. In GO and KEGG analysis, up-regulated CRRGs were significantly enriched at photosynthesis related terms (e.g. chloroplast organization, chlorophyll biosynthetic process, photosynthesis in GO, and porphyrin and chlorophyll metabolism in KEGG) and down-regulated CRRGs involved in stress response related terms (e.g. response to heat, protein folding, plant-type secondary cell wall biogenes, cell wall organization in GO; arginine and proline metabolism, amino sugar and nucleotide sugar metabolism, and carotenoid biosynthesis in KEGG) (**Supplementary Figure S1D, E**), which were different from CDRGs. Moreover, after re-watering, 1,030 (59%) upregulated CDRGs were downregulated. And 195 (11.2%) and 230 (13.2%) upregulated CDRGs were uniquely downregulated in AB and CD, respectively. For the downregulation of CDRGs, 838 (50.9%) genes were upregulated in the CRRGs, and 216 (13.1%) and 221 (13.4%) genes were specifically upregulated in AB and CD, respectively. Therefore, after re-watering, the growth of hybrids AB and CD was restored by resetting the drought response genes involved in photosynthesis and the stress response-related genes.

### Genes expression patterns in hybrids Zhengdan7137, Zhengdan7153, and their parental inbred lines

**Figure 4:**
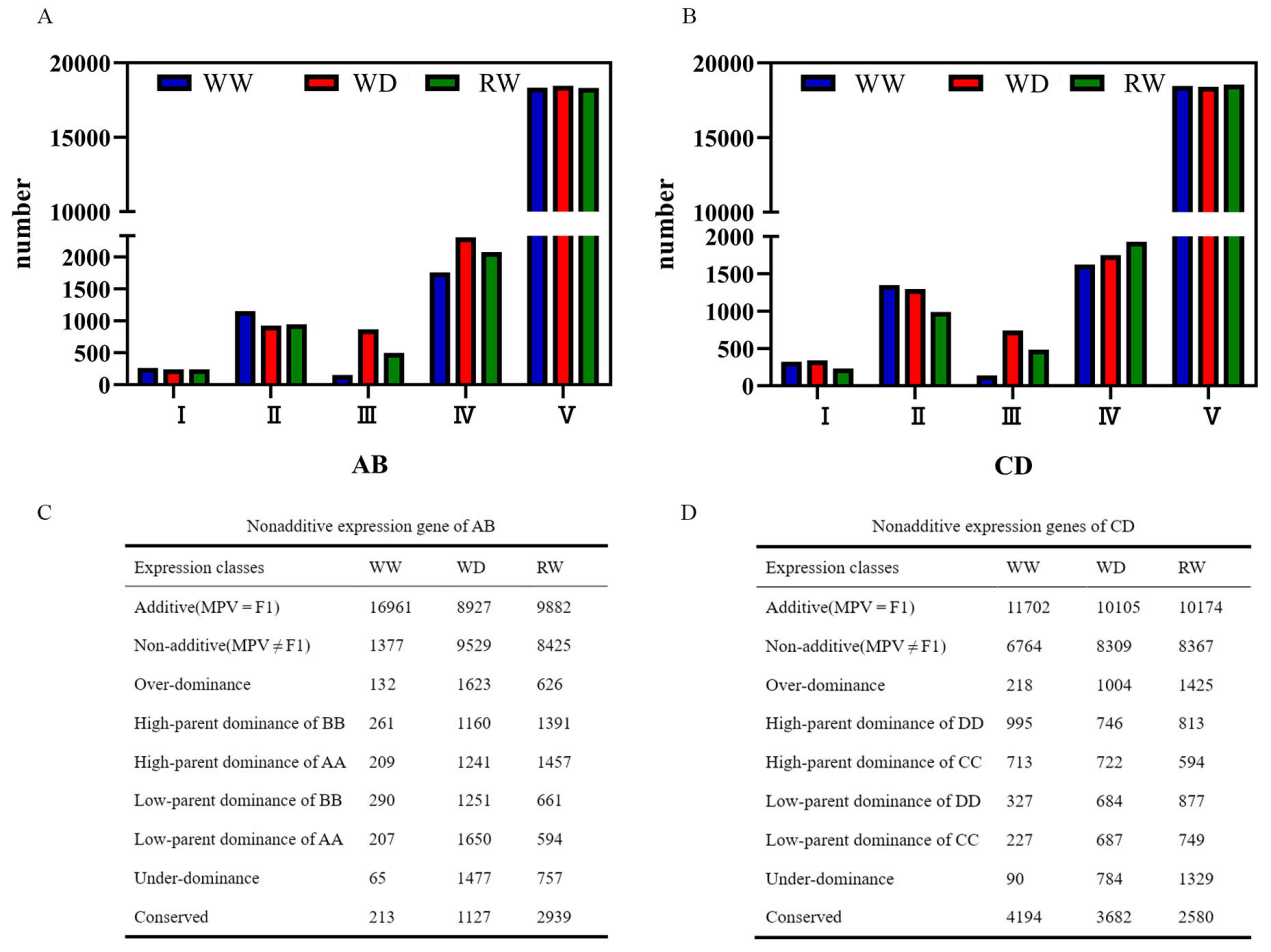
Genes expression patterns of hybrids Zhengdan7137 (AB) and Zhengdan7153 (CD) under well-watering (WW), water-deficited (WD) and re-watering (RW) conditions. **(A, B)** genes in PAV related expression patterns of AB **(A)** and CD **(B)**. AB and CD, Zhengdan7137 and Zhengdan7153. WW, WD and RW, well-watering, water-deficited and re-watering conditions; I, II, III, IV, and V were co-expression in both parents but not expression in hybrids genes (type I), only one parent specifically expressed genes (type II), hybrids specifically expressed genes (type III), hybrids and one specific parent expressed genes (type IV), and both parent and hybrid co-expression genes (type V); **(C, D)** nonadditive expression genes of AB **(C)** and CD **(D)**. AB, hybrid Zhengdan7137; AA, inbred line Zheng1110; BB, inbred line Zheng1117. AA and BB are parental inbred lines of AB. CD, hybrid Zhengdan7153; CC, inbred line Zheng1121; DD, inbred line Zheng641. CC and DD are parental inbred lines of CD.

To understand the mechanisms underlying the heterosis of hybrids AB and CD, we identified the DEGs between the hybrids (AB and CD) and their parental inbred lines and divided the DEGs into five types based on quality difference or presence/absence expression variation (PAV): co-expression in both parents but not expression in hybrids (type I), only one parent specifically expressed (type II), hybrids specifically expressed (type III), hybrids and one specific parent expressed (type IV), and both parent and hybrid co-expression (type V) (Zhao et al., 2019; Li et al., 2015). The number of type V genes was the highest (>81%) with few changes (18338_WW, 18456_WD, and 18307_RW in AB; 18456_WW, 18414_WD and 18541 RW in CD). Conversely, the number of type I genes was the lowest (264_WW, 242_WD, and 243_RW in AB; 324_WW, 339_WD, and 231_WD in CD). Type II and type IV genes slightly decreased and increased, respectively, when WD and RW conditions were compared with WW conditions. Interestingly, type III genes significantly increased under WD conditions (154_WW vs. 866_WD in AB and 137_WW vs. 740_WD in CD) and decreased under RW conditions (866_WD vs. 498_RW in AB and 740_WD vs. 483_RW in CD) (**Figures 4A, B**), which was consistent with the change in water potential.

Type V genes are further divided into additive and nonadditive genes. The additive genes were more abundant than nonadditive genes, except for AB under WD condition. The number of nonadditive genes increased under WD condition (9,529 in AB and 8,309 in CD) and RW condition (8,425 in AB and 8,367 in CD) when compared to WW condition (1,377 in AB and 6,764 in CD), especially for hybrid AB, in which nonadditive genes increased 6.9 and 6.1 times, respectively (**Figures 4C, D**). Nonadditive genes included overdominant, dominant, and underdominant genes. The quantity of overdominant and underdominant genes in AB and CD was less than the dominant expression genes under all WW, WD, and RW conditions (**Figures 4C, D**). Compared to WW condition, the numbers of overdominant, underdominant, and dominant genes were significantly increased under WD conditions, particularly for overdominant and underdominant genes which increased 4.6–22.7 times. After re-watering, the overdominant, underdominant, and dominant genes decreased by 61.4%, 48.7%, and 22.6% in AB and increased by 141.9%, 169.5%, and 106.8% in CD, respectively (**Figures 4C, D**). These results suggest that nonadditive genes responded to drought and re-watering stresses, especially the overdominant and underdominant genes, which was consistent with the drought-resistant heterosis of hybrids AB and CD.

### GO and KEGG analysis of overdominant and underdominant genes

Type III genes were essentially overdominant. Therefore, we combined them with the overdominant genes in type V and generated 286 (AB_WW), 355 (CD_WW), 2,489 (AB_WD), 1,744 (CD_WD), 1,124 (AB_RW), and 1,908 (CD_RW) overdominant genes. Under WW conditions, these overdominant genes were mainly enriched in photosynthesis-related terms in the GO and KEGG analyses (**Supplementary Figures S2A–D**). However, under WD conditions, the overdominant genes were significantly enriched in stress response-related terms such as response to heat, cell wall organization, plant hormone signal transduction, and phenylalanine, tyrosine, and tryptophan biosynthesis (**Supplementary Figures S3A–D**). Under RW conditions, the overdominant genes were recovered to enriched in the photosynthesis-related pathway. There were still many overdominant genes enriched in stress response-related terms, such as carotenoid biosynthesis in KEGG (**Supplementary Figures S4A–D**). We also identified 65 (AB_WW), 90 (CD_WW), 1,477 (AB_WD), 784 (CD_WD), 757 (AB_RW), and 1,329 (CD_RW) underdominant genes. Under WW conditions, different from overdominant genes, underdominant genes were enriched in stress-related terms in GO and KEGG analysis (e.g., flavonoid biosynthesis, phenylpropanoid biosynthesis, and phenylalanine metabolism in AB; response to heat, protein folding, and flavonoid biosynthesis in CD) (**Supplementary Figures S5A–D**). Under WD conditions, the underdominant genes were enriched during photosynthesis (e.g., photosynthesis in GO), antioxidant capacity (e.g., glutathione metabolism), and energy metabolism (e.g., pentose phosphate pathway and glycolysis/gluconeogenesis) (**Supplementary Figures S6A–D**). Under RW conditions, GO and KEGG analyses showed underdominant genes enriched in stress response-related processes and photosynthesis-related processes (e.g., response to heat, protein folding, response to stress and photosynthesis, and light harvesting in photosystem I in GO; photosynthesis-antenna proteins in KEGG) (**Supplementary Figures S7A–D**), which suggested that the physiological state of the hybrids was recovering. The variation in overdominance and underdominance genes under WD and RW conditions was similar to the upregulation and downregulation genes of CDRGs and CRRGs, which suggested that overdominant and underdominant genes might play important roles in dealing with drought and re-watering stresses.

### Conservative drought and re-watering response overdominance and underdominance genes

We identified common overdominant genes (714 and 722 under WD and RW conditions, respectively) and underdominant genes (584 and 492 under WD and RW conditions, respectively) between AB and CD. To gain further insight into the roles of over- and under-dominant genes in drought and re-watering stress resistance, we compared CDRGs and CRRGs with the common over- and under-dominant genes under WD and RW conditions, respectively. We found that the overdominant genes mainly overlapped with the upregulated CDRGs or CRRGs, whereas the underdominant genes mainly overlapped with the downregulated CDRGs or CRRGs. Under WD conditions, there were 303 overlapping genes between the upregulated CDRGs with common over-dominance genes and 252 overlapping genes between the downregulated CDRGs with common underdominance genes, designated as conservative drought response overdominance genes (DODGs) and underdominance genes (DUDGs). In addition, 165 and 267 common genes were identified by comparing unregulated and downregulated CRRGs to common overdominance and underdominance genes under RW conditions, respectively, and designated as conservative re-watering response overdominance genes (RODGs) and under-dominance expression genes (RUDGs).

**Figure 5:**
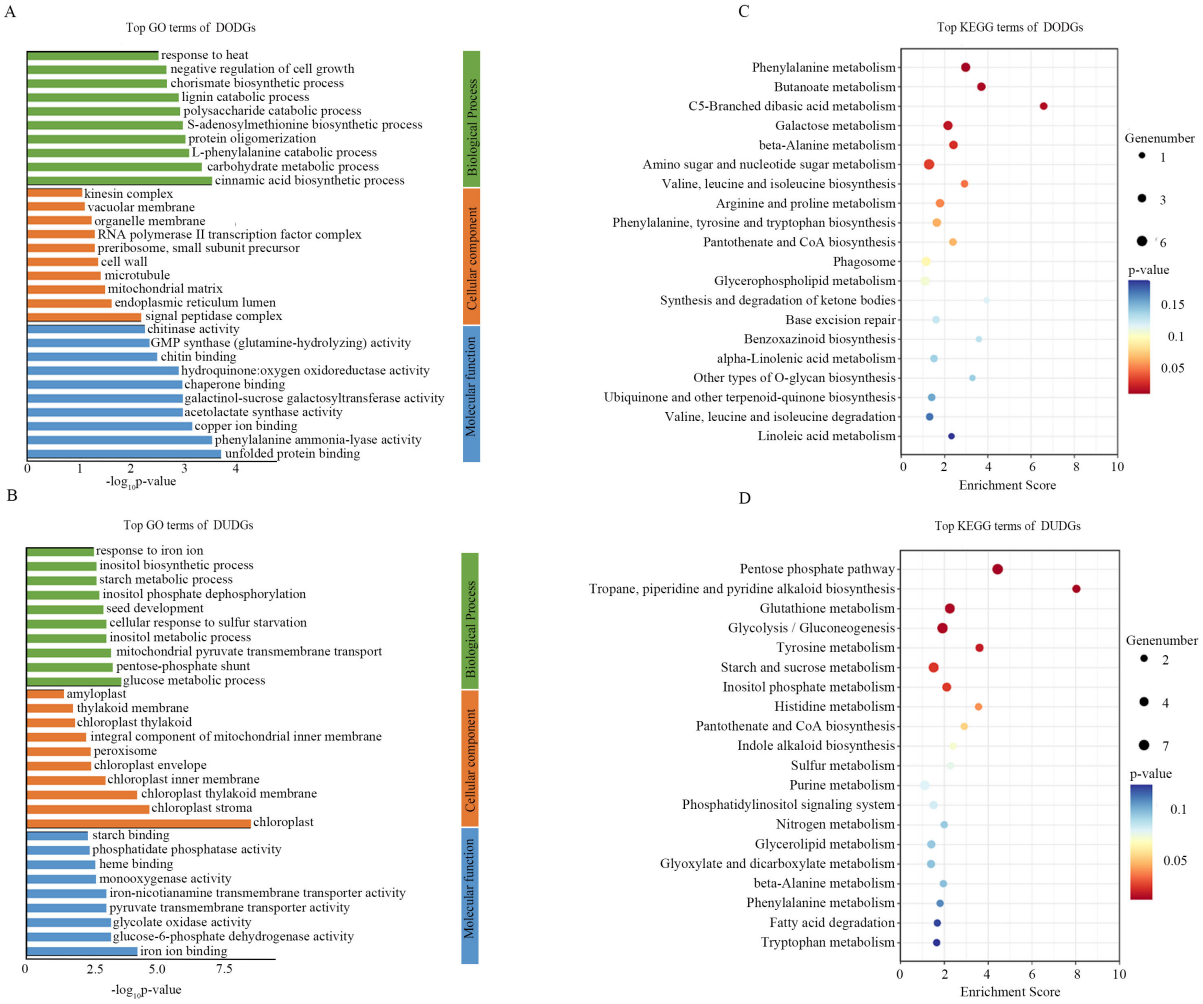
GO and KEGG analysis of DODGs and DUDGs. **(A, B)** the top 30 GO enrichment terms for DODGs and DUDGs; **(C, D)** the top 20 KEGG enrichment terms for DODGs and DUDGs.

The DODGs enriched at multiple stresses response processes and metabolism in GO and KEGG analysis, such as cinnamic acid biosynthetic process, carbohydrate metabolic process, L-phenylalanine catabolic process, S-adenosylmethionine biosynthetic process, and response to heat in GO; and phenylalanine metabolism, amino sugar and nucleotide sugar metabolism, and arginine and proline metabolism in KEGG (**Figures 5A, C**). DUDGs were enriched in the glucose metabolic process, pentose-phosphate shunt, starch metabolic process, pentose phosphate pathway, glutathione metabolism, and starch and sucrose metabolism (**Figures 5B, D**). In the RODGs, there were some cell propagation- and growth-related genes, as evidenced by their enrichment in the circadian rhythm, regulation of auxin biosynthetic processes, regulation of cyclin-dependent protein serine/threonine kinase activity, and photosynthesis in GO (**Supplementary Figures S8A, C**). In the RUDGs, stress-related terms such as response to heat, protein folding, and chaperone-mediated protein complex assembly were significantly enriched (**Supplementary Figures S8B, D**).

### qRT-PCR validation of overdominant genes

**Figure 6:**
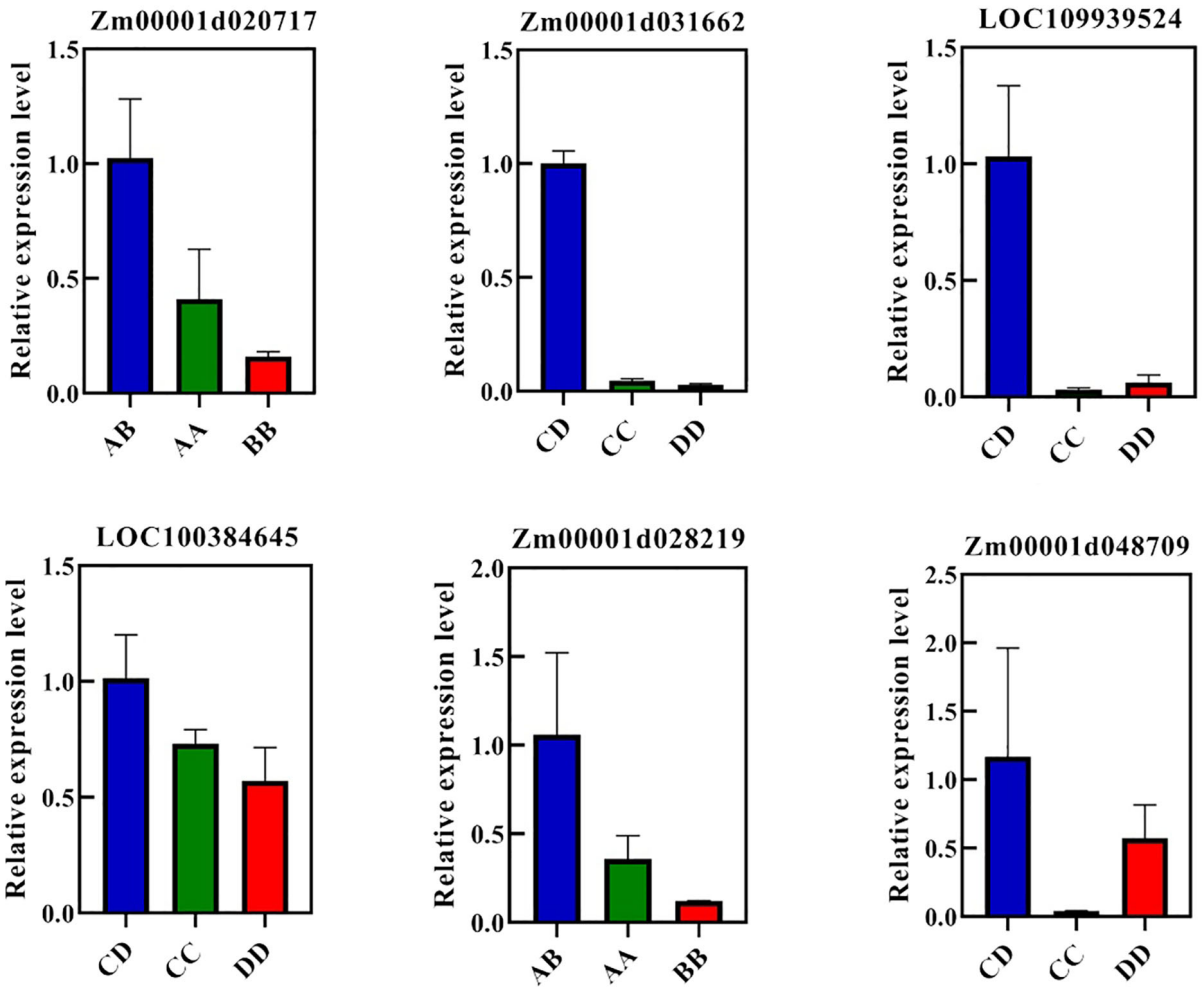
qRT-PCR for overdominant genes. *ZmGAPDH* was used as the internal reference. AB, hybrid Zhengdan7137; AA, inbred line Zheng1110; BB, inbred line Zheng1117. AA and BB are parental inbred lines of AB. CD, hybrid Zhengdan7153; CC, inbred line Zheng1121; DD, inbred line Zheng641. CC and DD are parental inbred lines of CD.

To validate the results of the RNA-Seq analysis, six overdominantly expressed DRGs or RRGs (Zm00001d020717, Zm00001d028219, Zm00001d048709, LOC100384645, LOC109939524, and Zm00001d031662) were randomly selected for quantitative real-time fluorescence polymerase chain reaction (qRT-PCR). Among these genes, Zm00001d028219 and Zm00001d020717 were DRG and RRG in AB, respectively, whereas Zm00001d048709 and the remaining genes (LOC100384645, LOC109939524, and Zm00001d031662) were DRG and RRGs in CD, respectively. The leaves of AB, CD, and their parental inbred lines were harvested under WD and RW conditions for qRT-PCR analysis. In qRT-PCR, the relative expression levels of these six genes were higher in the hybrids than in the parental inbred lines (**Figure 6**), which was consistent with the FPKM values in RNA-Seq and validated the reliability of our RNA-Seq data.

## Discussion

### Conservative mechanism of hybrids in response to drought and re-watering stresses

In natural agricultural environments, drought and re-watering often occur circularly for maize. When subjected to drought stress, plants trigger a series of physiological, biochemical, metabolic, and cellular responses to resist dehydration, including stress perception, signal transduction, activation or suppression of transcription factors, accumulation of osmoprotectants, protective proteins, and antioxidants, and stomatal closure, thereby reducing transpiration and photosynthesis (Sheoran et al., 2022). After re-watering, these responses can be restored to accelerate plant growth, depending on the plant species and intensity and duration of drought stress (Wang et al., 2022b). Therefore, drought resistance is not only related to the water scarcity response, but also to the re-watering response. However, systematic research focusing on the molecular mechanisms of drought and re-watering responses in maize is rare. In this study, we identified drought and re-watering response genes (DRGs and RRGs, respectively) in hybrids AB and CD and their parental inbred lines using transcriptomic analysis. Although there were large differences in DRGs and RRGs between the hybrids and their parental inbred lines, we found 3,411 (49.8% and 60.9%) and 3,133 (51.4% and 58.1%) conservative DRGs and RRGs (CDRGs and CRRGs) between AB and CD, respectively, indicating that there were conservative mechanisms of hybrids in response to drought and re-watering.

In CDRGs, the upregulation CDRGs was significantly enriched in stress response-related terms in GO and KEGG analyses, whereas the downregulation CDRGs was significantly enriched in photosynthesis-related terms exhibiting the injury of drought stress, which suggested that the upregulation of CDRGs played a predominant role in drought resistance. The most significantly enriched processes of up-regulation CDRGs in GO or KEGG analysis included cell wall biogenesis (plant-type primary and secondary cell wall biogenesis, cell wall organization, and cellulose biosynthesis), response to heat, trichome morphogenesis, L-phenylalanine metabolism (cinnamic acid biosynthesis and L-phenylalanine catabolic process), carbohydrate metabolism, and snRNA pseudouridine synthesis. Cell wall biogenesis, response to heat, L-phenylalanine metabolism, and carbohydrate metabolism have been recognized to contribute to drought resistance in many studies (Li et al., 2024; Waititu et al., 2021; Zhang et al., 2022b; Amara et al., 2013; Wang et al., 2023). Stiffening and thickening of the cell wall increase the strength of plant cells to resist high tension caused by water deficiency, reduce water loss, and enhance drought resistance (Alvarez et al., 2008). The heat response involves heat shock proteins (HSPs) and late embryogenesis abundant proteins (LEAs), which are two important types of protective proteins that respond to diverse abiotic stresses (Wang et al., 2023; Ristic et al., 1991). Overexpression of the LEA proteins *Rab28* and *ZmNHL1* improves drought resistance in transgenic maize plants (Amara et al., 2013; Wang et al., 2023). Augmentation of the L-phenylalanine metabolism pathway is a universal strategy for plants to resist abiotic stress by producing more antioxidants such as flavonoids and anthocyanins (Yuan et al., 2022; Adams et al., 2019). Carbohydrate metabolism increases the concentration of soluble sugars (e.g., sucrose, amino sugars, and nucleotide sugars) as osmoprotectants to resist drought stress (Fang et al., 2024). Although trichomes may reduce transpiration under water scarcity conditions and pseudouridine modification of snRNA is involved in the response to stress (Xuan et al., 2020; Lippman and Zamir, 2007), their function in drought resistance in plants has not been well investigated.

After re-watering, GO and KEGG analyses of upregulated CRRGs showed that photosynthesis was robustly recovered, which was the basis for hybrids to compensate or overcompensate for the losses caused by drought stress. Downregulation of CRRGs is involved in many processes that are upregulated under drought stress, such as responses to heat, cell wall biogenesis (plant-type secondary cell wall biogenesis and cell wall organization), benzoxazinoid biosynthesis, amino sugar and nucleotide sugar metabolism, and arginine and proline metabolism. Downregulation of these drought response processes contributes to the adaptation of maize to the recovered water potential and reduced growth obstacles. In addition, 1,225–1,260 (70.2–72.2%) upregulated CDRGs and 1,054–1,059 (64.0–64.3%) downregulated CDRGs in AB or CD were recovered after re-watering, indicating that these genes were adjusted with water potential and might play key roles in resisting drought and re-watering stress. Yet, there were still 498–462 (28.5–26.5%) upregulated CDRGs and 555–519 (33.7–31.5%) in AB or CD that did not change after re-watering, which might be the reason for pre-exposure to drought enhancing drought resistance as drought stress memory (Ding et al., 2012).

### Nonadditive expression genes conservatively contribute to hybrid heterosis under drought stress

In previous studies, nonadditive genes in hybrids that were significantly different from the average of their parents were deemed responsible for heterosis (Zhao et al., 2019). In this study, we investigated the gene expression patterns of the maize hybrids Zhengdan7137(AB) and Zhengdan7153(CD) under well-watered, water-deficit, and recover-watered conditions. AB and CD were obtained from SS×NSS and Reid×Tangsipingtou heterotic patterns, respectively. These two heterotic patterns are widely used in China, especially in the major production areas Huanghuaihai. Therefore, the molecular mechanisms of heterosis in AB and CD are representative in China. Under well-watering conditions, there were 1,377 (7.5%) and 6,764 (36.7%) nonadditive genes in AD, and CD, respectively. Under water-deficit conditions, the number of nonadditive genes increased to 9,529 (51.6%) and 8,390 (45.4%) in AB and CD, respectively. The phenomenon that the percentage of nonadditive genes increased under abiotic stress was also observed in previous studies. For examples, Zheng et al. identified 46.5–52.6% and 57.6–62.08% nonadditive genes in maize reciprocal hybrids Zhengdan538 under control and water-deficit conditions, respectively (Zhang et al., 2022a). There are 2,326 (10.5%) and 5,817 (26.2%) nonadditive genes in the hybrid An’nong591 under controlled conditions and heat stress, respectively (Zhao et al., 2019). Therefore, it is usual for hybrids to increase nonadditive genes under abiotic stresses, which may be favorable for hybrids to resist environmental stresses. Under re-watering conditions, nonadditive gene slightly decreased in hybrids AB (8,425, 46%) and CD (8,367, 45.1%) compared to water-deficit conditions. This suggests that re-watering was incapable of completely eliminating the influence of drought on the gene expression patterns of the hybrids, which might be a mechanism by which hybrids maintain drought memory.

Nonadditive genes included overdominant, dominant, and underdominant genes. Compared to the dominant genes, the numbers of overdominant and underdominant genes were more sensitive to water deficit and re-watering conditions in hybrids AB and CD. Under well-watering conditions, over- and under-dominant genes were significantly enriched in photosynthesis-related and stress response-related processes, respectively. Overdominant expression of photosynthesis-related genes might provide a higher photosynthetic capacity for hybrids, which, in turn, provides more photosynthetic products to promote growth than their parental inbred lines. The elevated expression of stress-response genes represses plant growth and reduces grain yield (Sun et al., 2023). The under-dominant expression of stress response genes in hybrids may minimize their obstacle effects on growth. Therefore, these over- and under-dominant genes may contribute to the rapid growth of hybrids, which are important for heterosis under WW conditions. In contrast to well-watered conditions, overdominant genes were significantly enriched in stress response-related processes under water-deficit conditions, whereas underdominant genes were significantly enriched in photosynthesis-related processes. In addition, we identified 714 and 584 common over-dominant and under-dominant genes, respectively, between hybrids AB and CD. Approximately 43% of these overdominant and underdominant genes were upregulated and downregulated CDRGs, respectively, and were designated as DODGs and DUDGs, respectively. DODGs and DUDGs are enriched in multiple resistance processes, such as l-phenylalanine metabolism, carbohydrate metabolism, and response to heat and glutathione metabolism. These results indicate that increased over-dominance and under-dominance of genes under WD conditions enhanced the drought resistance of hybrids. After re-watering, overdominant genes and RODGs were significantly enriched in cell propagation processes, photosynthesis-related processes, and stress response-related processes, and under-dominant genes and RUDGs were enriched in stress response-related processes, which accelerated the recovery of hybrids from drought stress and retained drought stress memory. Therefore, it was a conservative mechanism by which hybrids adjusted their overdominance and underdominance genes to deal with drought and re-watering stresses, which contributed to hybrid heterosis under drought stress.

### Conservative drought response overexpression genes are potential targets for drought resistance improvement

Drought is a destructive abiotic stress that leads to significant maize yield losses. Although many drought-resistance genes have been cloned in maize, novel and excellent gene resources are required to improve maize drought resistance without yield losses. Considering that upregulated drought response genes play a dominant role in drought resistance and that overdominant genes are important for heterosis under drought stress, we suggest that DODGs involved in multiple pathways are valuable potential targets for improving drought resistance in maize. Drought stress signals are detected in the plasma membrane and activate successive signal transduction pathways. Ca^2+^ is a secondary messenger that responds to drought stress (Jiang et al., 2013). In DODGs, there are several genes involved in Ca^2+^ signaling pathways; for example, LOC100273477 and LOC100141385 each encode a calcium-binding protein annexin and a CBL-interacting protein kinase (CIPK), which are involved in Ca^2+^ signal perception and activation of downstream drought response proteins (Ma et al., 2020; Saad et al., 2019). In addition, protein kinases (e.g., MAP kinase protein LOC100278992, calcium-dependent protein kinase LOC100383301, and serine/threonine protein kinase LOC100384302) may be involved in drought signal transduction (Chen et al., 2021). The drought signal is then transduced into the nucleus to regulate the expression of transcription factors. We identified 27 transcription factors from 15 families in DODGs, such as five MYB, three NAC, three bHLH, three trihelix, two ERF, and one bZIP, which fine-tune the expression of downstream genes to deal with drought stress (Singh et al., 2023; Mao et al., 2015; Wei et al., 2021; Cao et al., 2021; Xie et al., 2019; Zhao et al., 2023). Polyamines (PAs) consisting of putrescine, spermidine, and spermine promote reactive oxygen species scavenging and stabilize macromolecules and membrane structures to resist abiotic stress. Arginine and methionine are precursors for PA biosynthesis (Shi and Chan, 2014). Among the DODGs, arginine decarboxylase (AY110562) and S-adenosylmethionine synthase (c12757_5c) are the two key rate-limiting enzymes that catalyze the conversion of L-arginine and methionine into putrescine (Shi and Chan, 2014). Additionally, polyamine oxidase (PAO1 and LOC103632539) might enhance PA content (Liu et al., 2022). Shikimate kinase phosphorylates shikimate into 3-phosphate shikimate in the shikimate pathway and promoted L-phenylalanine, anthocyanin and flavonoid synthesis (Yuan et al., 2022). Phenylalanine ammonia lyase (PAL) catalyzes the conversion of l-phenylalanine to trans-cinnamic acid, which is the key rate-limiting step in phenylpropanoid biosynthesis (Zhang et al., 2022b). Phenylpropanoids are further catalyzed into flavonoids and anthocyanins, which function as antioxidants (Yuan et al., 2022). There was one shikimate kinase (LOC10019326) and three PALs (PAL13, LOC100285115, and LOC103653804) in DODGs. Moreover, anthocyanidin 3-O-glucosyltransferase (LOC100282556) catalyzes the formation of the first stable anthocyanin (Wang et al., 2022a). Thus, antioxidant (e.g., flavonoid and anthocyanin) production chain centered on L-phenylalanine metabolism was enhanced under water-deficit conditions. Lignin is another downstream product of l-phenylalanine metabolism and is important for cell wall biogenesis (Zhan et al., 2022; Pesquet et al., 2024). Furthermore, cellulose synthase CESA8, Brittle stalk-2-like proteins BK2L3/7, tubulin Tua5/Tub3/TUBB5, and microtubule-associated proteins (MAP65/70) in DODGs may participate in the remodeling of cell wall properties under water scarcity (Julius et al., 2021; Wang et al., 2022c; Sasaki et al., 2023). Increased plant tissue temperature and cellular viscosity under drought stress cause protein denaturation, which is detrimental to plant physiological and biochemical reactions. HSPs and LEAs are two important types of protective proteins in response to diverse abiotic stresses (Wang et al., 2023; Ristic et al., 1991). We identified nine HSPs (e.g., LOC100286044, LOC103653979, and LOC100282976) and three LEAs (DHN1, LOC100279027, and LOC103643070) in the DODGs. DODGs are also involved in sucrose and hexosamine biosynthesis, glycolysis, lysine catabolism, and amino sugar metabolism. Hence, verifying the functions of these DODGs in drought resistance is urgently required.

## Conclusions

Understanding the inheritance and molecular mechanisms of drought-resistance heterosis will advance the improvement and breeding of maize plants with high drought resistance and yield. Our findings identified conservative drought and re-watering stress response genes, indicating that nonadditive genes, especially overdominant and underdominant genes, conservatively contributed to hybrid heterosis under drought stress and suggested that DODGs are potential targets for drought resistance improvement. These results revealed the conserved molecular mechanisms of drought resistance and heterosis of hybrids, which were helpful for drought resistance improvement.

## Methods

### Material planting and drought treatments

The hybrids Zhengdan7137(AB), Zhengdan7153(CD), and their parental inbred lines [Zheng1110(AA), Zheng1117(BB), Zheng1121(CC), and Zheng641(DD)] were sown on June 3th in two parallel blocks at 5m×0.6m×0.15m in the experimental fields of the Henan Academy of Agricultural Sciences, located in Yuanyang, China (113.7° E, 35° N). Standard irrigation and fertilizer management were performed to ensure seed germination. After seed germination, one block was not irrigated until the relative humidity of soil at the depth of 10-20cm (R) ≦ 30%, i.e. circularly suffered water-deficit (WD, 30%< R ≦ 60%) treatment and re-watering treatment (RW, R≥60%). The other block irrigated normally, as well-watering (WW, R≥60%) treatment. In VT stage, three plants of AB, CD, and their parental inbred lines under the WW, WD, and RW treatment were randomly selected, and ear leaves were harvested and stored at -80°C until use.

### cDNA library preparation

Total RNA of harvested leaves from WW, WD, and RW treatments was extracted from each sample using the TRIzol reagent (Invitrogen, CA, USA). RNA degradation and contamination of RNAs was tested using 1% agarose gel electrophoresis. RNA quality of RNAs were assessed using a Bioanalyzer 2100 system (Agilent Technologies, Santa Clara, CA, USA) with a minimum RNA integrity index (RIN) of 7.0. High-quality RNA was selected for cDNA library construction. Libraries were prepared using the VAHTS Universal V6 RNA-Seq library preparation kit, according to the manufacturer’s instructions.

### cDNA libraries sequencing and RNA-Seq data analysis

The RNA-Seq libraries were sequenced on an Illumina NovaSeq 6000 platform, generating 150 base-pair paired-end reads. Initially, raw reads in the Fastq format underwent quality control using the fastp tool to remove low-quality reads and yield clean reads (Chen et al., 2018). The clean reads were aligned to the reference genome available at (ftp://ftp.ncbi.nlm.nih.gov/genomes/all/GCF/000/005/005/GCF_000005005.2_B73_RefGen_v4/GCF_000005005.2_B73_RefGen_v4_genomic.fna.gz)by using HISAT2 (Kim et al., 2015). Only reads exhibiting a perfect match or a single mismatch were retained for further analysis. To quantify gene expression, the FPKM values for each gene were computed and the read counts for each gene were determined using HTSeq-count (Anders et al., 2015). To assess the consistency of the biological replicates, Pearson correlation rates were calculated using R software (version 3.2.0). Differential expression analysis was performed using DESeq2 ((|fold change| > 2 and FDR < 5%) to identify differentially expressed genes (DEGs) (Love et al., 2014). GO and KEGG pathway analyses of the DEGs were performed using R (v 3.2.0) (The Gene Ontology C, 2019; Kanehisa et al., 2008).

### Classification of gene expression patterns

First, we categorized the differentially expressed genes (DEGs) based on pairwise comparisons between the hybrids (AB and CD) and their two parental inbred lines, we categorized the DEGs into five types according to their expression patterns under WW, WD, and RW conditions (Zhao et al., 2019). The five types were both parental inbred lines expressing but silent in hybrid genes (type I, P1, P2>0&F_1_ = 0), one specific parental inbred line expressing genes (type II, P1>0&P2, F_1_ = 0 or P2>0&P1, F_1_ = 0), the hybrid specifically expressing genes (type III, F_1_>0&P1, P2 = 0), the hybrid and one specific parental inbred line expressing genes (type IV, P1, F_1_>0&P2 = 0 or P2, F_1_>0&P1 = 0) and both parental inbred lines and the hybrid co-expressing genes (type V, P1, P2, F1>0) (Zhao et al., 2019, 2019). Subsequently, type V genes were further divided into additive genes (F_1_=MPV, MPV=(P1+P2)/2) and nonadditive genes (F_1_≠MPV). Nonadditive contained over-dominant (F_1_>P1, P2), under-dominant genes (F_1_<P1, P2), high dominance (F_1_=P1&P1>P2 or F_1_=P2&P2>P1), and low dominance genes (F_1_=P1&P1<P2 or F_1_=P2&P2<P1).

### Quantitative real-time fluorescence polymerase chain reaction

Quantitative real-time fluorescence polymerase chain reaction (qRT-PCR) was used to validate the expression of six dominant genes. qRT-PCR was performed using a CFX96 Real-Time PCR Detection System. The transcript levels of each gene were measured using a 25 µL volume of SYBR (Accurate Biology, Changsha, China) on a CFX96 real-time PCR assay system (Bio-Rad, Hercules, CA, USA). qRT-PCR thermal cycling treatments included a single pre-incubation at 95°C for 30 s, followed by 5 s at 95°C and 56°C for 30 s for 40 amplification cycles, a 95°C ramp-up and a melt curve of 65 to 95°C with an increment of 0.5°C. GAPDH was used as an internal control. Primers used are listed in **Supplementary Table 3**.

### Statistical analysis

We measured the grain weight, ear length, and ear thickness of every 5 years with 3 repeats. The grain moisture content was measured in two replicates using a PM-8188-A moisture meter (Kett Electric Laboratory, Tokyo, Japan). Phenotypic data were analyzed using SPSS (version 26; SPSS Inc., Chicago, IL, USA) and Excel (version 2021; Excel Inc., Chicago, IL, USA). To assess significant differences between means, the Student’s t-test and Duncan’s multiple comparison test were employed. Additionally, we calculated mid-parent heterosis (MPH) and high-parent heterosis (HPH) to evaluate heterosis between hybrids and parents. The MPH was determined using the formula: MPH (%) = (F_1_ - MP)/MP, where MP represented the parents’ mean. Additionally, HPH was calculated using the formula: HPH (%) = (F_1_ - HP)/HP, where HP was the high parent.

## Data Availability

The raw data supporting the conclusions in this article are available in the BioProject database under accession number PRJNA1107710 (http://www.ncbi.nlm.nih.gov/bioproject/1107710).
