# Supplementary material for: Molecular mechanisms of heterosis under drought stress in maize hybrids Zhengdan7137 and Zhengdan7153
Source: Front Plant Sci. 2024 Oct 8;15:1487639. doi: 10.3389/fpls.2024.1487639 (PMC11494150; doi:10.3389/fpls.2024.1487639)
Supplement: Supplementary file 1 [file DataSheet1.docx]

**
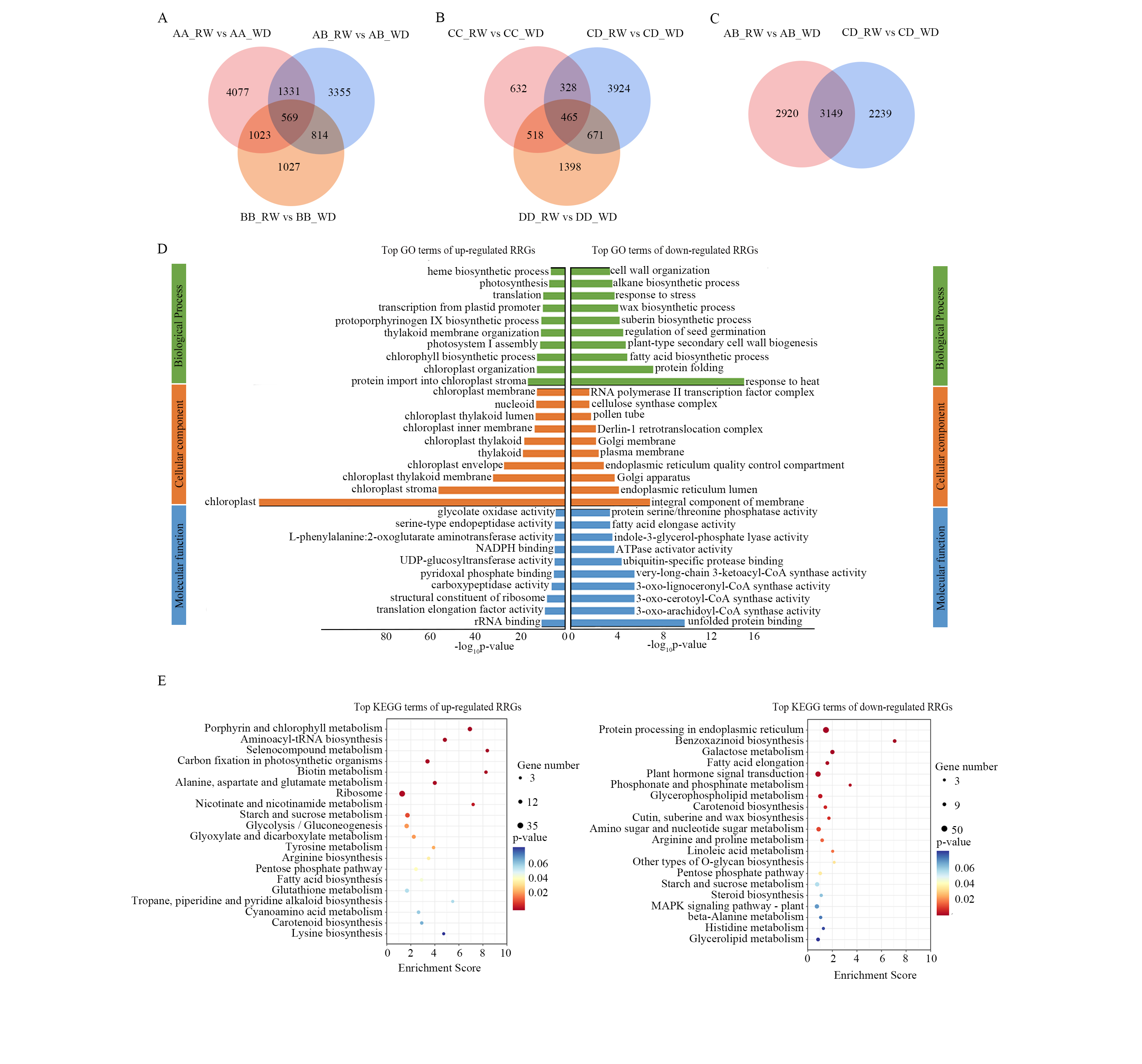
**

**Supplementary Figure S1** Re-watered response genes of AB, CD and their parental inbred lines. **(A)** and **(B)**,venn diagrams of re-watered response genes in AB, AA and BB **(A)**, and CD, CC and DD **(B)**; **(C)**, venn diagrams of re-watered response genes of AB and CD; (D), top 30 enrichment terms in GO analysis of up- and down- regulated CRRGs; **(E)**, top 20 enrichment terms in KEEG pathways analysis of up- **(left)** and down- **(right)** regulated CRRGs. AB, hybrid Zhengdan7137; AA, inbred line Zheng1110; BB, inbred line Zheng1117. AA and BB are parental inbred lines of AB. CD, hybrid Zhengdan7153; CC, inbred line Zheng1121; DD, inbred line Zheng641. CC and DD are parental inbred lines of CD.


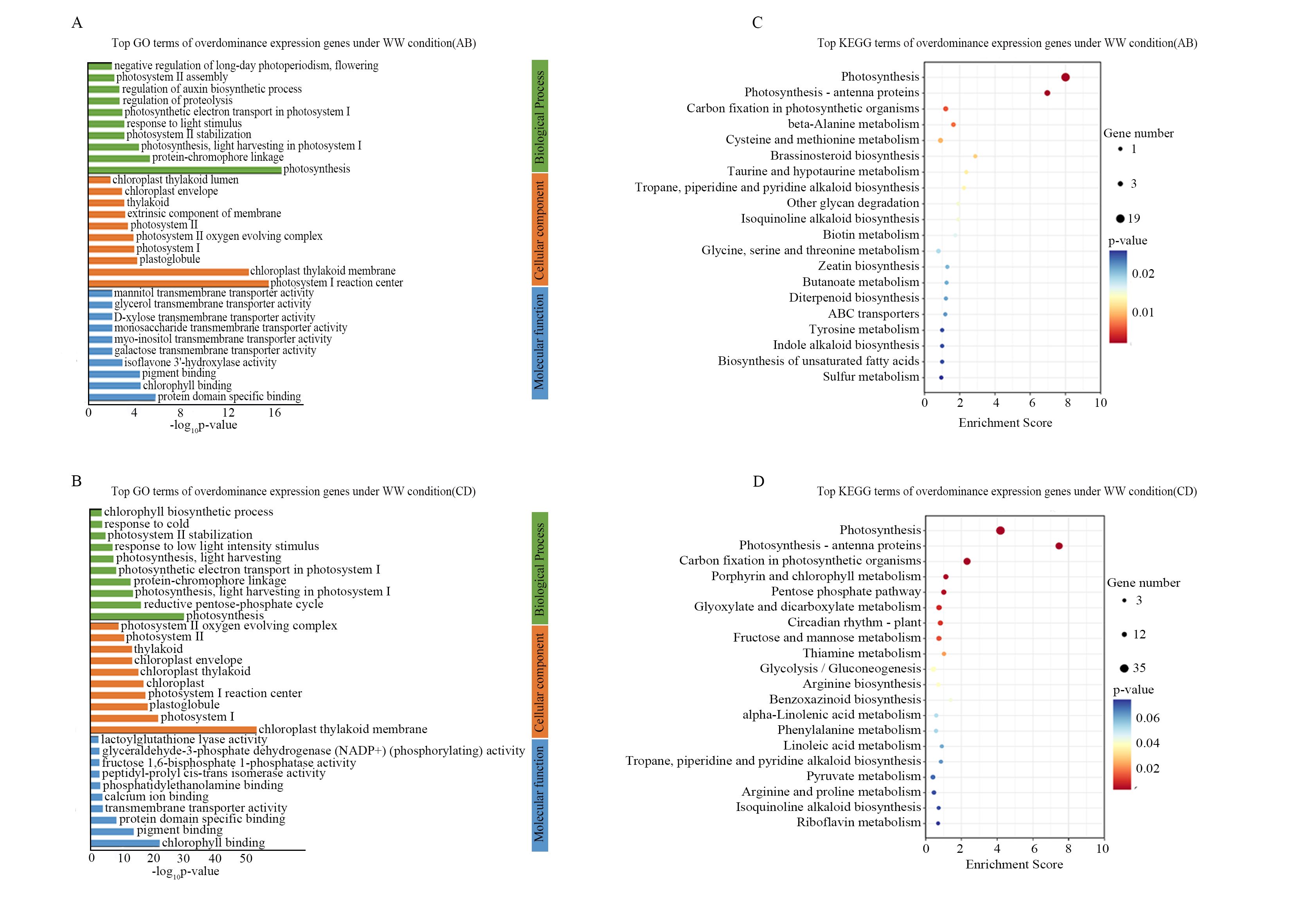


**Supplementary Figure S2** GO and KEGG of overdominance expression genes of AB and CD under WW condition. **(A)** and **(B)**, the top 30 GO terms of AB **(A)** and CD **(B)** under WW condition. **(C)** and **(D)**, the top 20 KEGG pathways of AB **(C)** and CD **(D)** under WW condition. “AB” and “CD”, hybrids Zhengdan7137 and Zhengdan7153.


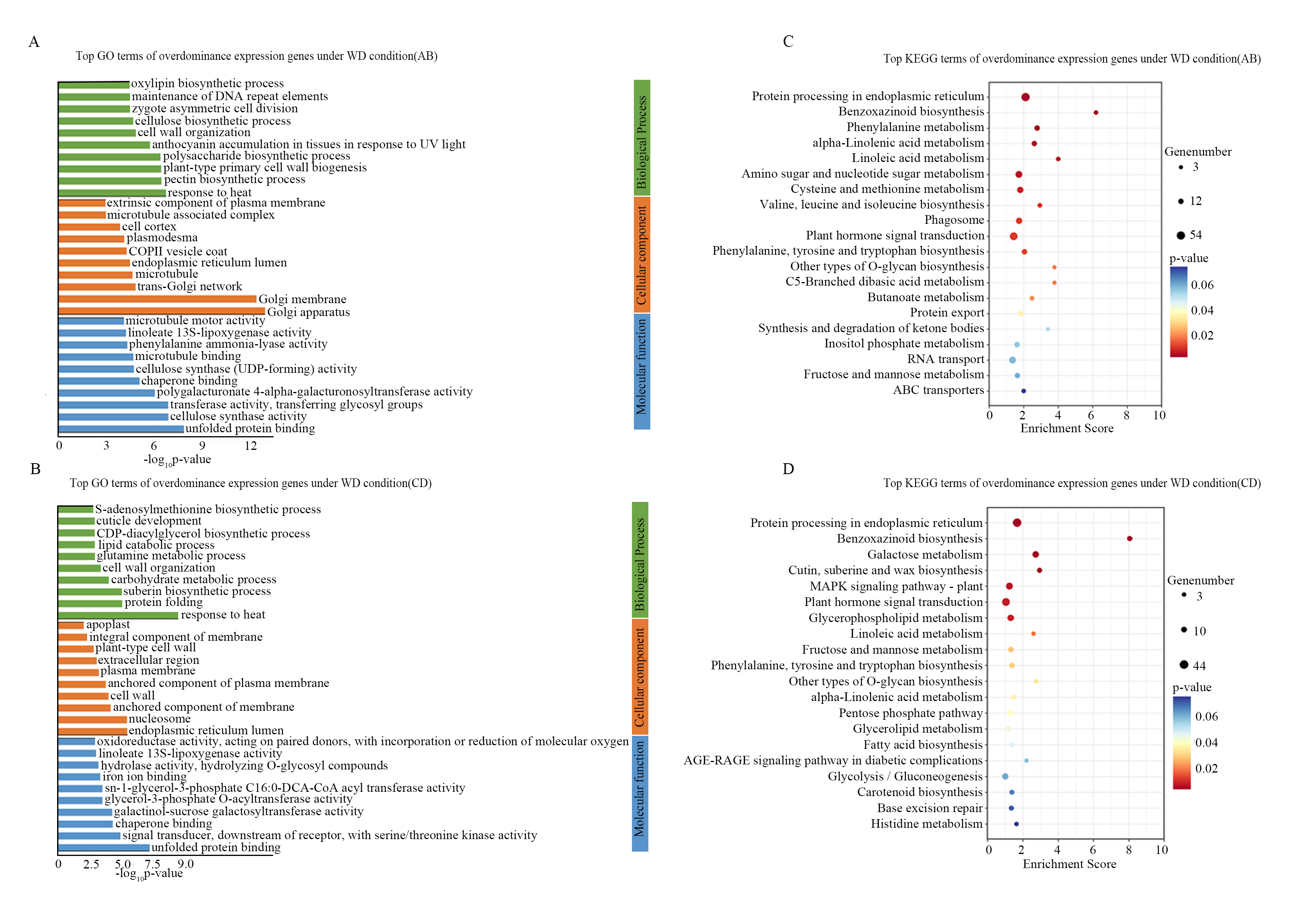


**Supplementary Figure S3** GO and KEGG of overdominance expression genes of AB and CD under WD condition. **(A)** and **(B)**, the top 30 GO terms of AB **(A)** and CD **(B)** under WD condition. **(C)** and **(D)**, the top 20 KEGG pathways of AB **(C)** and CD **(D)** under WD condition. “AB” and “CD”, hybrids Zhengdan7137 and Zhengdan7153.


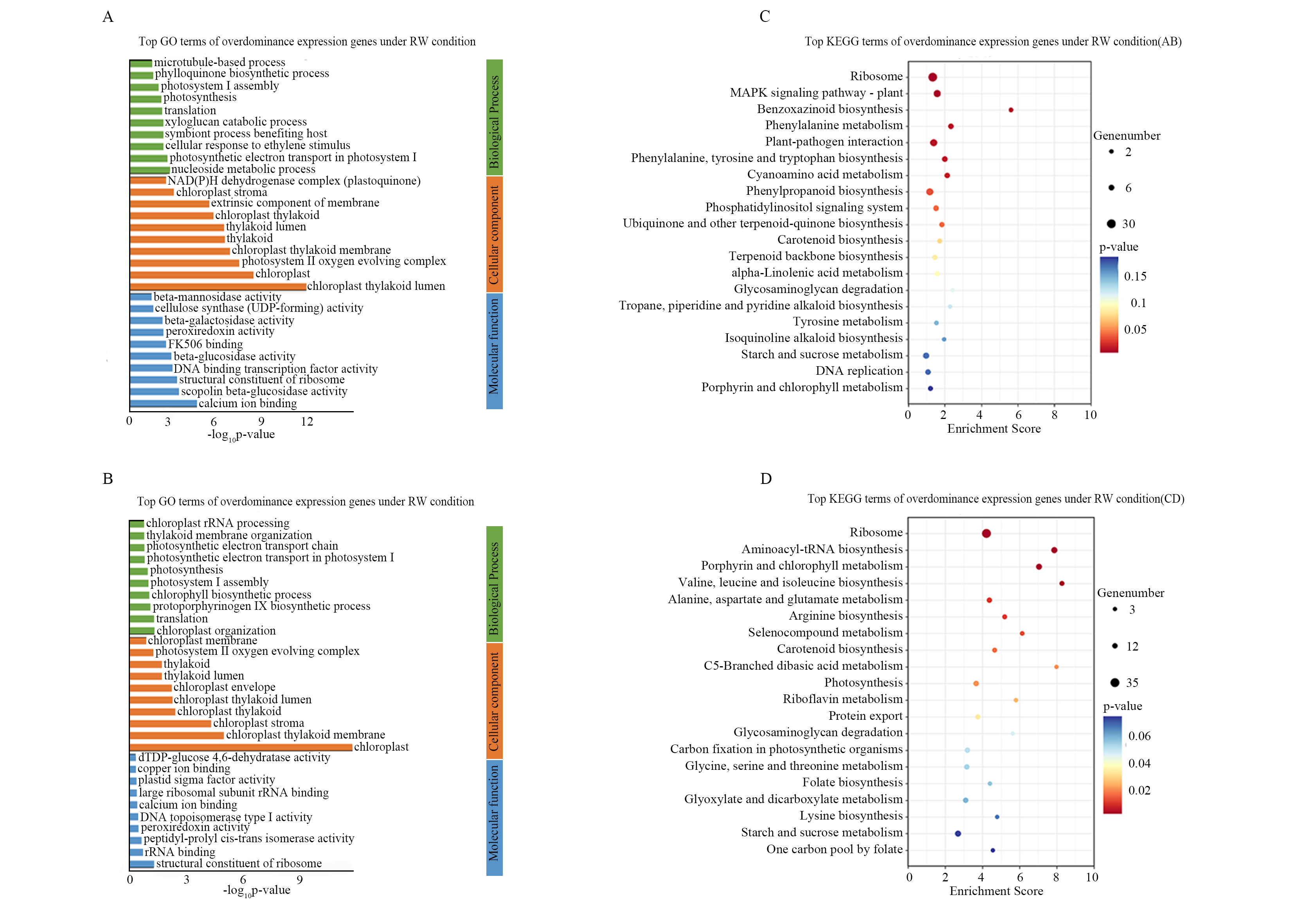


**Supplementary Figure S4** GO and KEGG of overdominance expression genes of AB and CD under RW condition. **(A)** and **(B)**, the top 30 GO terms of AB **(A)** and CD **(B)** under RW condition. **(C)** and **(D)**, the top 20 KEGG pathways of AB **(C)** and CD **(D)** under RW condition. “AB” and “CD”, hybrids Zhengdan7137 and Zhengdan7153.


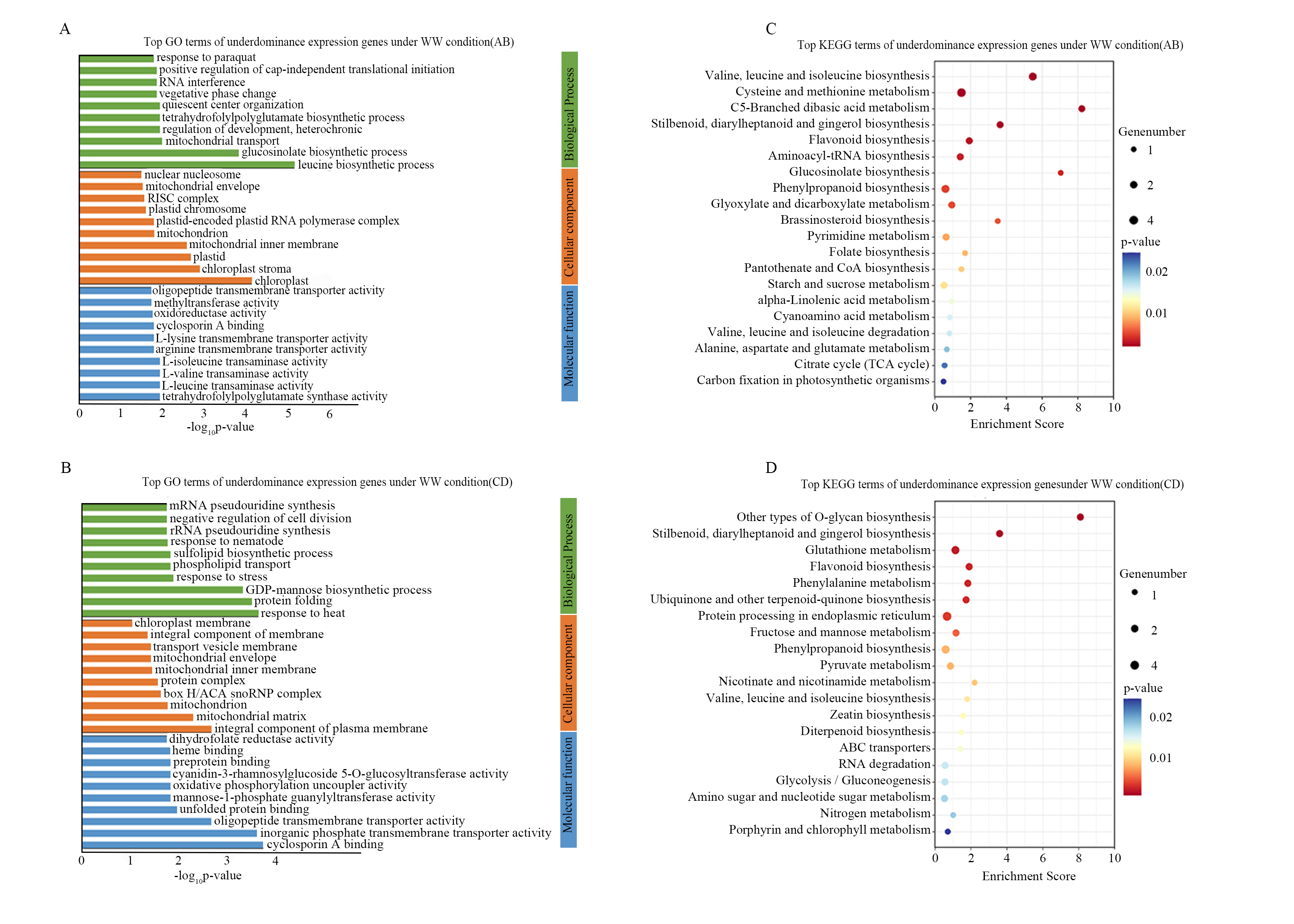


**Supplementary Figure S5** GO and KEGG of underdominance expression genes of AB and CD under WW condition. **(A)** and **(B)**, the top 30 GO terms of AB **(A)** and CD **(B)** under WW condition. **(C)** and **(D)**, the top 20 KEGG pathways of AB **(C)** and CD **(D)** under WW condition. “AB” and “CD”, hybrids Zhengdan7137 and Zhengdan7153.


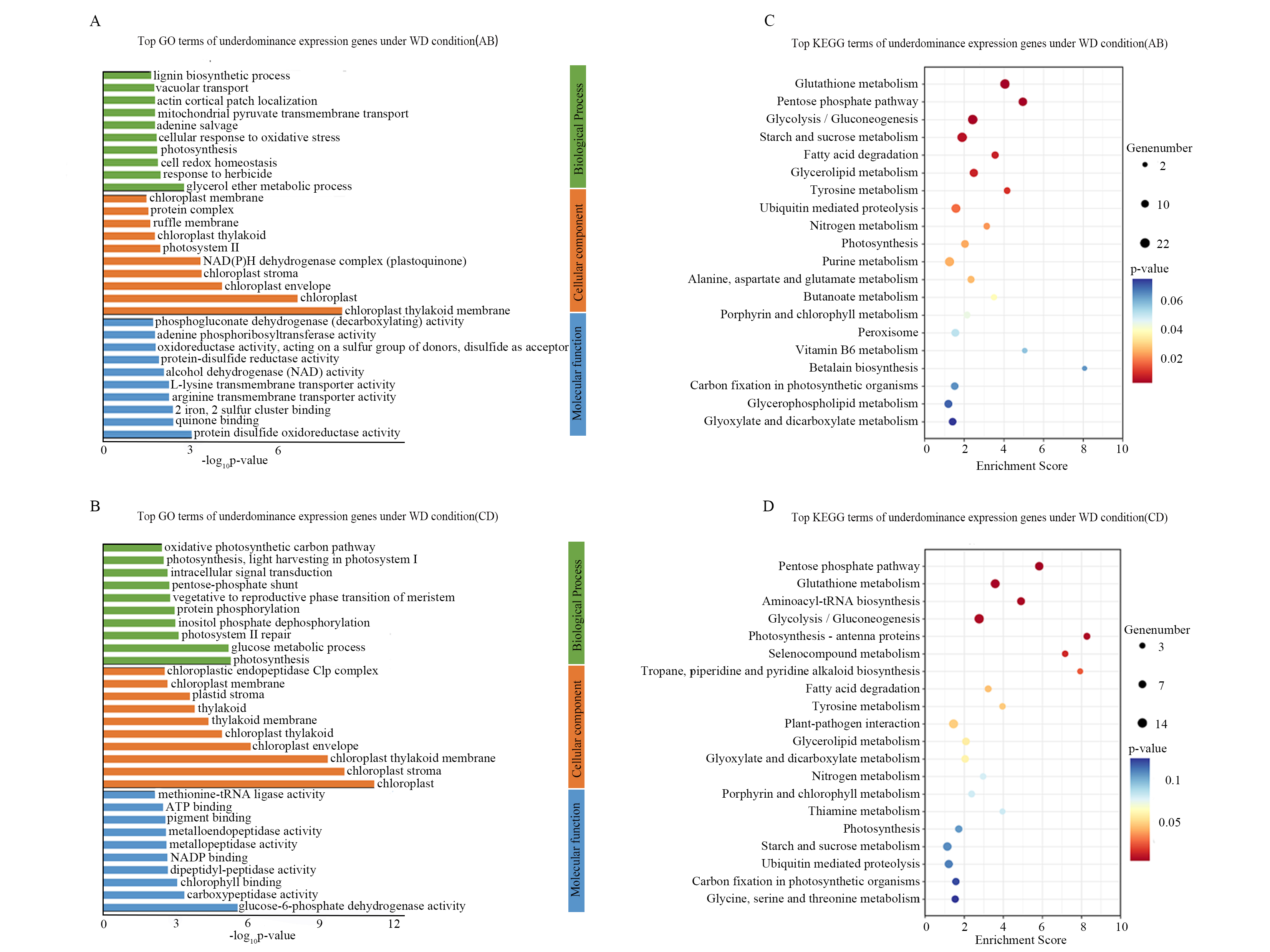


**Supplementary Figure S6** GO and KEGG of underdominance expression genes of AB and CD under WD condition. **(A)** and **(B)**, the top 30 GO terms of AB **(A)** and CD **(B)** under WD condition. **(C)** and **(D)**, the top 20 KEGG pathways of AB **(C)** and CD **(D)** under WD condition. “AB” and “CD”, hybrids Zhengdan7137 and Zhengdan7153.


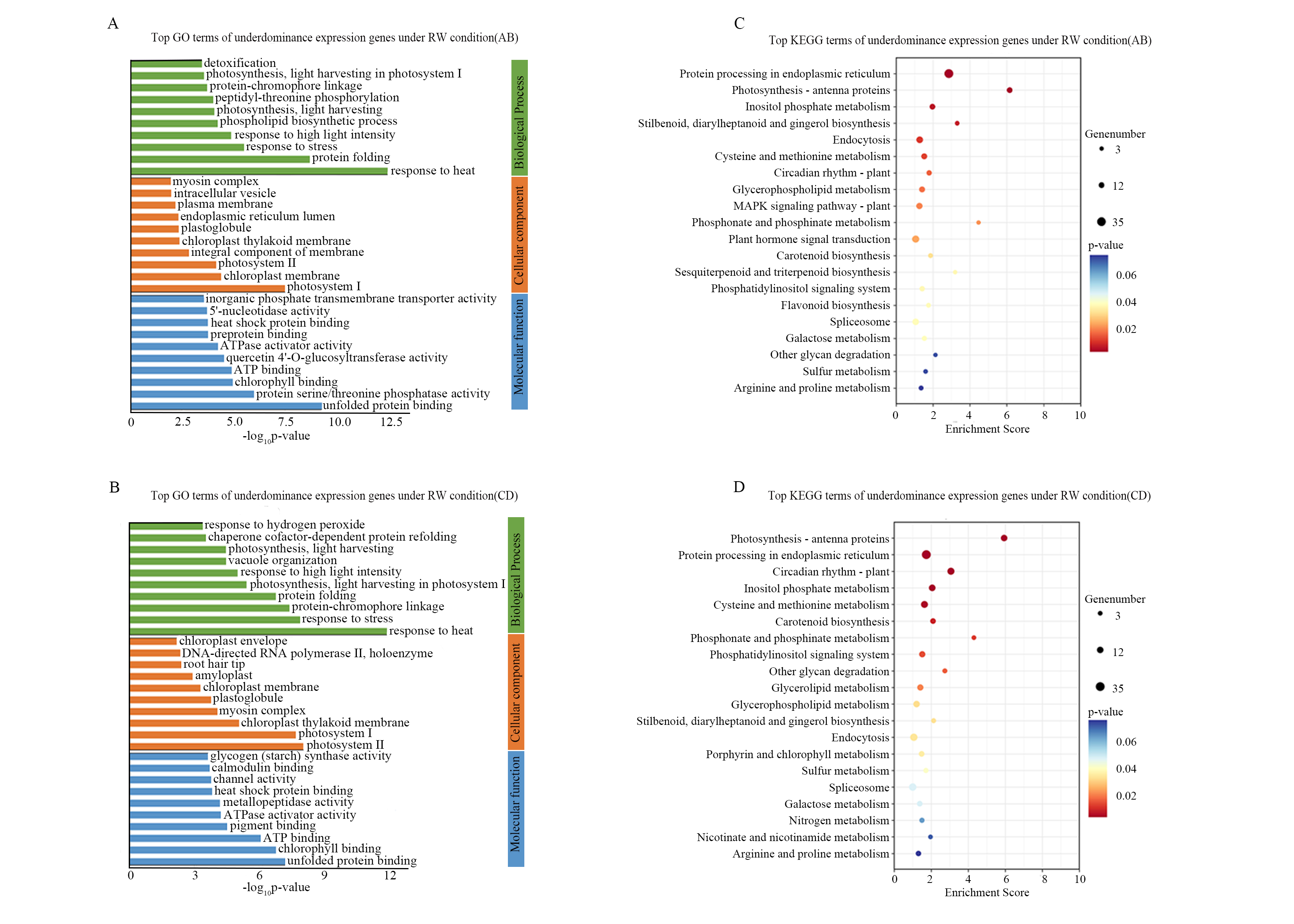


**Supplementary Figure S7** GO and KEGG of underdominance expression genes of AB and CD under RW condition. **(A)** and **(B)**, the top 30 GO terms of AB **(A)** and CD **(B)** under RW condition. **(C)** and **(D)**, the top 20 KEGG pathways of AB **(C)** and CD **(D)** under RW condition. “AB” and “CD”, hybrids Zhengdan7137 and Zhengdan7153.


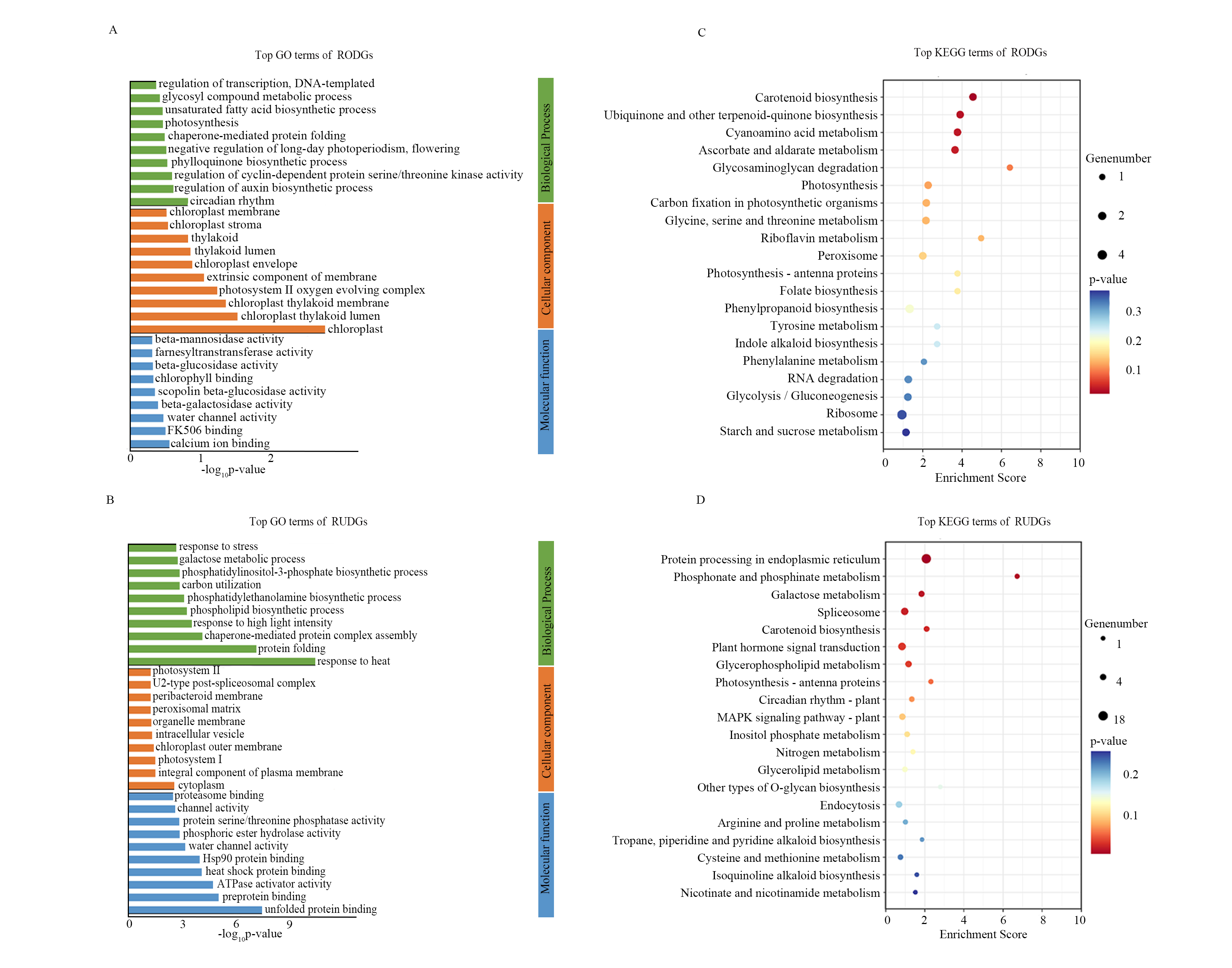


**Supplementary Figure S8** GO and KEGG analysis of RODGs and RUDGs. **(A)** and **(B)**, the top 30 GO enrichment terms for RODGs and RUDGs; **(C)** and **(D)**, the top 20 KEGG enrichment terms for RODGs and RUDGs. “AB” and “CD”, hybrids Zhengdan7137 and Zhengdan7153.

**Supplementary Table S1** Ear component traits of hybrid AB, CD and their parental inbred lines under WW and WD conditions.

| Ear traits | Treatments | AB | AA | BB | MPH | HPH |
| --- | --- | --- | --- | --- | --- | --- |
| EL (cm) | WW | 16.2±1.18a | 13.2±0.21b | 10.8±0.17c | 35.0% | 22.7% |
|  | WD | 15.0±2.77a | 11.2±0.35b** | 11.0±0.10b | 35.1% | 33.9% |
| ET (cm) | WW | 4.4±0.02a | 4.0±0.21b | 3.8±0.11c | 12.8% | 10.0% |
|  | WD | 4.4±0.25a | 3.2±0.00b** | 3.4±0.00b** | 33.3% | 29.4% |
| KW (g) | WW | 32.7±0.49a | 29.3±3.92a | 24.6±1.95b | 21.3% | no. |
|  | WD | 35.5±1.19a* | 22.0±2.11b* | 22.6±1.79b | 59.2% | 57.1% |
| GY (g) | WW | 104.6±9.37a | 54.1±5.57b | 53.7±6.99c | 94.1% | 93.3% |
|  | WD | 99.4±3.61a | 27.8±4.96b** | 38.9±0.66b* | 198.1% | 155.5% |
| KWC (%) | WW | 19.6±0.23a | 18.7±0.31ab | 18.0±1.23b | 6.8% | no. |
|  | WD | 17.0±0.58a** | 15.2±0.53b* | 15.3±0.62b* | 11.5% | 11.1% |
| Ear traits | Treatments | CD | CC | DD | MPH | HPH |
| EL (cm) | WW | 15.6±0.36a | 13.2±0.11b | 11.7±1.06c | 25.30% | 18.2% |
|  | WD | 15.5±0.67a | 10.8±0.37b** | 10.7±0.70b | 44.19% | 43.5% |
| ET (cm) | WW | 4.5±0.08a | 3.7±0.06c | 3.9±0.11b | 18.42% | 15.4% |
|  | WD | 4.5±0.25a | 3.4±0.23b | 3.5±0.15b* | 30.43% | 28.6% |
| KW (g) | WW | 31.4±0.83a | 27.8±0.51b | 23.1±1.09c | 23.38% | 12.9% |
|  | WD | 37.2±3.22a* | 36.1±1.23a** | 24.6±2.03b | 22.57% | no. |
| GY (g) | WW | 109.8±10.95a | 39.1±3.66c | 59.2±1.12b | 123.40% | 85.5% |
|  | WD | 101.4±9.58a | 34.7±5.35c* | 42.9±6.28b* | 161.34% | 136.4% |
| KWC (%) | WW | 20.5±1.08a | 17.7±0.92b | 19.4±0.25a | 10.51% | no. |
|  | WD | 18.1±0.53a* | 15.7±0.58b* | 15.9±0.38b* | 14.56% | 13.8% |

AB, AA and BB, hybrid Zhengdan7137 and their parental inbred lines Zheng1110 and Zheng1117; CD, CC and DD, hybrid Zhengdan7153 and their parental inbred lines Zheng1121 and Zheng641; MPH, mid-parent heterosis; HPH, high-parent heterosis; EL, ear length; ET, ear thickness; GY, ear grain yield; KW, 100 kernels weight; KWC, kernels water content; a, b and c, the p-value < 0.05 among hybrid and their parental inbred lines in Duncan multiple range test; no., no HPH. “ * ”, p-value < 0.05 between WW and WD conditions in student’s t-test.

**Supplementary Table S2** The RNA-seq sequencing data of AB, CD and their parental inbred lines under WW, WD and RW conditions

| sample | Raw Reads  (M) | Raw Bases  (G) | Clean  Reads  (M) | Clean Bases  (G) | Q30  (%) | GC(%) | Uniquely mapped  (%) |
| --- | --- | --- | --- | --- | --- | --- | --- |
| AA_WW1 | 42.78 | 6.42 | 42.4 | 6.33 | 92.75 | 54.56 | 89 |
| AA_WW2 | 41.74 | 6.26 | 41.41 | 6.19 | 92.81 | 54.75 | 88.5 |
| AA_WW3 | 43.65 | 6.55 | 43.3 | 6.47 | 92.99 | 54.33 | 88 |
| AA_RW1 | 43.66 | 6.55 | 43.36 | 6.48 | 91.9 | 54.04 | 87.8 |
| AA_RW2 | 39.37 | 5.91 | 39.11 | 5.84 | 93.43 | 54.01 | 88.5 |
| AA_RW3 | 43.78 | 6.57 | 43.47 | 6.48 | 93.48 | 54.23 | 88.4 |
| AA_WD1 | 45.32 | 6.8 | 44.99 | 6.7 | 92.05 | 53.12 | 55.4 |
| AA_WD2 | 44.93 | 6.74 | 44.64 | 6.66 | 92.06 | 52.47 | 81.7 |
| AA_WD3 | 46.92 | 7.04 | 46.68 | 6.93 | 93.07 | 51.93 | 74.6 |
| AB_WW1 | 43.88 | 6.58 | 43.53 | 6.5 | 93.08 | 54.97 | 87.7 |
| AB_WW2 | 39.45 | 5.92 | 39.15 | 5.84 | 92.66 | 55.78 | 88.1 |
| AB_WW3 | 42.09 | 6.31 | 41.78 | 6.24 | 93.19 | 54.26 | 83.7 |
| AB_RW1 | 43.4 | 6.51 | 43.16 | 6.45 | 92.31 | 53.65 | 80.1 |
| AB_RW2 | 44.55 | 6.68 | 44.24 | 6.6 | 93.17 | 53.97 | 86.8 |
| AB_RW3 | 45.82 | 6.87 | 45.52 | 6.8 | 91.91 | 53.68 | 87.1 |
| AB_WD1 | 39.78 | 5.97 | 39.51 | 5.89 | 91.91 | 53.36 | 87.3 |
| AB_WD2 | 42.79 | 6.42 | 42.56 | 6.36 | 92.76 | 53.74 | 87.6 |
| AB_WD3 | 41.94 | 6.29 | 41.7 | 6.23 | 92.11 | 53.41 | 87 |
| BB_WW1 | 43.5 | 6.53 | 43.17 | 6.45 | 92.83 | 53.86 | 86.5 |
| BB_WW2 | 42.54 | 6.38 | 42.26 | 6.31 | 92.72 | 54.11 | 86.2 |
| BB_WW3 | 42.93 | 6.44 | 42.61 | 6.36 | 93.19 | 54.07 | 85.8 |
| BB_RW1 | 42.3 | 6.35 | 41.98 | 6.26 | 93.27 | 54.26 | 86.1 |
| BB_RW2 | 43.1 | 6.47 | 42.76 | 6.38 | 93.03 | 54.24 | 85 |
| BB_RW3 | 43.1 | 6.46 | 42.77 | 6.39 | 93.22 | 53.83 | 84.7 |
| BB_WD1 | 44.29 | 6.64 | 44.02 | 6.58 | 92.26 | 54.49 | 86.3 |
| BB_WD2 | 44.12 | 6.62 | 43.87 | 6.56 | 92.2 | 54.36 | 82.7 |
| BB_WD3 | 41.2 | 6.18 | 40.95 | 6.12 | 92.01 | 55.09 | 82.6 |
| CC_WW1 | 45.08 | 6.76 | 44.74 | 6.69 | 92.99 | 54.11 | 85 |
| CC_WW2 | 39.77 | 5.96 | 39.5 | 5.9 | 93.39 | 53.67 | 88 |
| CC_WW3 | 47.45 | 7.12 | 47.07 | 7.03 | 93.21 | 54.26 | 88.2 |
| CC_RW1 | 45.9 | 6.88 | 45.66 | 6.81 | 92.6 | 53.6 | 87.8 |
| CC_RW2 | 51.34 | 7.7 | 50.02 | 7.46 | 92.39 | 53.02 | 88 |
| CC_RW3 | 46.74 | 7.01 | 46.46 | 6.93 | 92.25 | 54.05 | 88 |
| CC_WD1 | 41.8 | 6.27 | 41.53 | 6.21 | 92.15 | 54.6 | 87 |
| CC_WD2 | 45.19 | 6.78 | 44.93 | 6.71 | 92.36 | 54.36 | 86.9 |
| CC_WD3 | 43.97 | 6.6 | 43.67 | 6.53 | 91.83 | 53.86 | 86.5 |
| CD_WW1 | 39.68 | 5.95 | 39.39 | 5.88 | 93.15 | 54.56 | 84.2 |
| CD_WW2 | 43.48 | 6.52 | 43.2 | 6.43 | 93.22 | 54.8 | 86 |
| CD_WW3 | 40.46 | 6.07 | 40.15 | 5.99 | 93.03 | 55.02 | 84.4 |
| CD_RW1 | 45.7 | 6.85 | 45.46 | 6.79 | 92.28 | 53.91 | 86.9 |
| CD_RW2 | 45.52 | 6.83 | 45.25 | 6.76 | 92.5 | 54.26 | 85.8 |
| CD_RW3 | 42.48 | 6.37 | 42.22 | 6.29 | 92.03 | 53.4 | 86.9 |
| CD_WD1 | 44.41 | 6.66 | 44.12 | 6.59 | 91.9 | 54.08 | 86.6 |
| CD_WD2 | 43.61 | 6.54 | 43.34 | 6.47 | 92.36 | 54.51 | 86.7 |
| CD_WD3 | 42.96 | 6.44 | 42.69 | 6.38 | 92.03 | 53.9 | 86.9 |
| DD_WW1 | 46.08 | 6.91 | 45.73 | 6.82 | 93.18 | 53.72 | 84.7 |
| DD_WW2 | 42.77 | 6.42 | 42.43 | 6.34 | 92.65 | 54.21 | 85.9 |
| DD_WW3 | 47.29 | 7.09 | 46.95 | 7.02 | 93.05 | 53.6 | 85.9 |
| DD_RW1 | 43.42 | 6.51 | 43.14 | 6.44 | 91.8 | 53.97 | 87.3 |
| DD_RW2 | 50.05 | 7.51 | 47.18 | 7.05 | 92.09 | 54.51 | 87.1 |
| DD_RW3 | 43.23 | 6.48 | 42.92 | 6.41 | 91.85 | 54.49 | 87.5 |
| DD_WD1 | 46.46 | 6.97 | 46.17 | 6.88 | 92.14 | 53.97 | 86.7 |
| DD_WD2 | 39.66 | 5.95 | 39.45 | 5.88 | 92.5 | 53.19 | 86.5 |
| DD_WD3 | 42.09 | 6.31 | 41.86 | 6.23 | 92.68 | 53.81 | 86.8 |

**Supplementary Table S3** Primers for qRT-PCR

| Gene ID | Primer direction | Primer sequence (5’‐3’) |
| --- | --- | --- |
| ZmGAPDH/LOC542367 | F | F:ATCAACGGCTTCGGAAGGAT |
|  | R | R:CCGTGGACGGTGTCGTACTT |
| Zm00001d048709 | F | F:CAACACCAGCCATACCAG |
|  | R | R:CATACTCCTCCAGCCTCC |
| LOC100384645 | F | F:TTAATTCGCTGTGGTGT |
|  | R | R:TTTGTTCCGATACTTGC |
| LOC109939524 | F | F:CACATCTTGGTCGGTCAT |
|  | R | R:CGATTCTTGGGATTTCTT |
| Zm00001d031662 | F | F:TACCGATCTTATCTCCCA |
|  | R | R:ACCTTCCGTTTGTTGC |
| Zm00001d020717 | F | F:TGTTCAAGCCGACCTACC |
|  | R | R:CTCAGCACCCGATTCATT |
| Zm00001d028219 | F | F:CGATTTGATAGCCCTGGAA |
|  | R | R:ACGTGCCTTGGAATTTGG |
